# 40S Ribosome Biogenesis Co-Factors Are Essential for Gametophyte and Embryo Development

**DOI:** 10.1371/journal.pone.0054084

**Published:** 2013-01-30

**Authors:** Sandra Missbach, Benjamin L. Weis, Roman Martin, Stefan Simm, Markus T. Bohnsack, Enrico Schleiff

**Affiliations:** 1 Department of Biosciences, Molecular Cell Biology of Plants, Goethe University, Frankfurt/Main, Germany; 2 Cluster of Excellence Frankfurt; Goethe University, Frankfurt/Main, Germany; 3 Center of Membrane Proteomics, Goethe University, Frankfurt/Main, Germany; Université Libre de Bruxelles, Belgium

## Abstract

Ribosome biogenesis is well described in *Saccharomyces cerevisiae*. In contrast only very little information is available on this pathway in plants. This study presents the characterization of five putative protein co-factors of ribosome biogenesis in *Arabidopsis thaliana*, namely Rrp5, Pwp2, Nob1, Enp1 and Noc4. The characterization of the proteins in respect to localization, enzymatic activity and association with pre-ribosomal complexes is shown. Additionally, analyses of T-DNA insertion mutants aimed to reveal an involvement of the plant co-factors in ribosome biogenesis. The investigated proteins localize mainly to the nucleolus or the nucleus, and atEnp1 and atNob1 co-migrate with 40S pre-ribosomal complexes. The analysis of T-DNA insertion lines revealed that all proteins are essential in *Arabidopsis thaliana* and mutant plants show alterations of rRNA intermediate abundance already in the heterozygous state. The most significant alteration was observed in the *NOB1* T-DNA insertion line where the P-A3 fragment, a 23S-like rRNA precursor, accumulated. The transmission of the T-DNA through the male and female gametophyte was strongly inhibited indicating a high importance of ribosome co-factor genes in the haploid stages of plant development. Additionally impaired embryogenesis was observed in some mutant plant lines. All results support an involvement of the analyzed proteins in ribosome biogenesis but differences in rRNA processing, gametophyte and embryo development suggested an alternative regulation in plants.

## Introduction

Ribosome biogenesis requires the coordination of roughly 200 protein co-factors that assist in 60S and 40S subunit assembly and ribosomal RNA (rRNA) processing [1]–[4]. The maturation of ribosomal subunits initiates with transcription of the 35S pre-rRNA by RNA polymerase I in the nucleolus, which is gradually cleaved to generate the mature 18S, 5.8S and 25S rRNAs. The 5S rRNA associated with the 60S subunit is independently transcribed by RNA polymerase III [5]. In plants, investigations of pre-rRNA processing indicate the conservation of the overall cleavage sites [6]–[9], but differences to the processing pathway in yeast cannot be excluded since not all cleavage sites on plant rRNA have been mapped so far.

Only few factors involved in ribosome biogenesis in plants have been characterized. On the one hand, plant homoloques to yeast proteins like the eukaryotic translation initiation factor 6 (eIf6), the exoribonuclease 2 (Xrn2) or rRNA processing co-factors nucleolar complex associated protein 1 (Noc1)/maintenance of killer 21 (Mak21) have been identified [8]–[11]. On the other hand, one plant-specific protein-family was identified, for which a function in ribosome biogenesis is suggested [12]. *Domino1* homozygous deletion mutants arrest early in embryogenesis in the globular stage and show enlarged nucleoli in the embryo and the endosperm. Based on this and subsequent studies it is assumed that alterations in nucleolar structure and defects in embryogenesis are phenotypes associated with impaired ribosome biogenesis [13]–[15]. Thus, the ribosome biogenesis pathway appears to be an ideal subject for investigation of embryogenesis and defects thereof caused by malfunction of factors involved.

In angiosperms embryogenesis starts with the double fertilization of the ovule with two sperm cells delivered by one pollen tube [16]–[18] resulting in different copies of male and female genomes in the cells of one seed. One sperm cell fuses with the egg cell to form the zygote and the other merges with the diploid central cell resulting in the formation of the endosperm which provides the nutrients for the developing embryo. Additional maternal tissue of the ovule surrounds the embryo and endosperm and later forms the seed coat [19]. Due to the high impact of the maternal tissue on embryo development [20]–[21] most embryo lethal phenotypes are the consequence of defective female gametophyte development and function [22]. Additionally a variety of mutations are known that affect the male gametophyte [23]. These mutations mainly lead to an inability of the pollen to form pollen tubes [24]–[25] or impair meiosis and mitosis of the microspores [26]–[27]. The corresponding gene products causing these defects when mutated or missing are involved in a variety of pathways and so far no cellular process could be identified where malfunction of all components lead to disturbed male gametophyte development.

Female and male gametophyte phenotypes are similar in reduced transmission of the mutated allele. In fully-penetrating female gametophyte-specific mutations no transmission through the female gametophyte takes place and therefore the sporophytic generation cannot be homozygous [28]. These aberrant transmission rates do not follow Mendelian segregation patterns, as observed by backcrossing of a heterozygous female with a WT male or *vice versa* [28]. Apart from an aberrant segregation the homozygous individuals can be arrested in different stages of embryo development [29].

**Figure 1:**
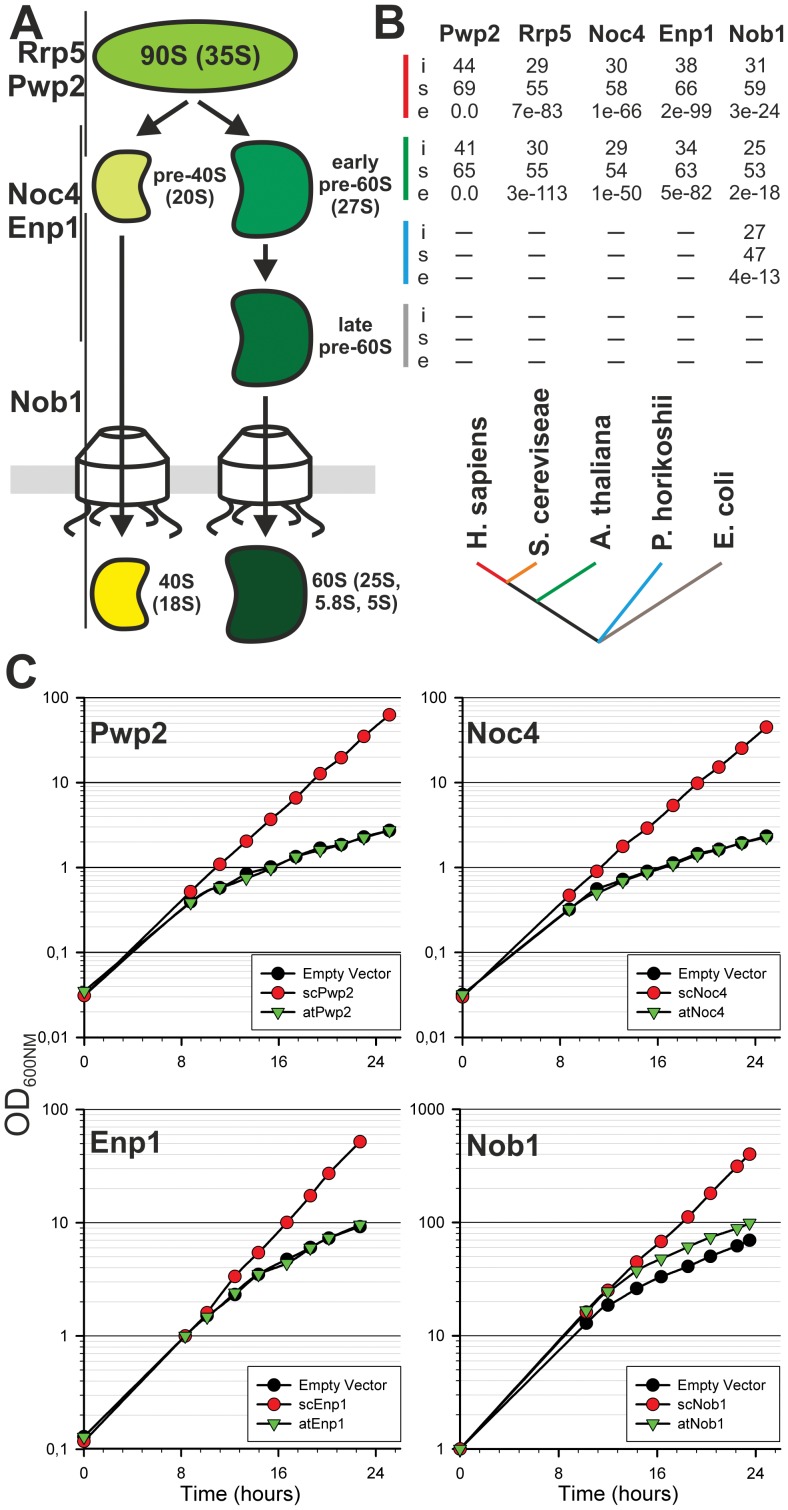
Evolutionary distribution and functional conservation of selected factors. A, Ribosome biogenesis starts with a 90S precursor which is processed to the 40S and 60S subunit. Association of the proteins during maturation of the 40S is indicated. B, The yeast factors were used as bait to perform a forward and reversed Blast search. The *S. cerevisiae* sequences were compared with the *H. sapiens* (red), *A. thaliana* (green), *P. hirokoshii* (blue) and *E. coli* (grey). Values for identity and similarity (in percentage; i and s, respectively) between bait and sequence identified in the corresponding species (indicated by color as in the phylogenetic scheme), and the e-value of the Blast search is given (e). A dash indicates that no sequence was identified fulfilling the criteria. C, Growth curve of *Saccharomyces cerevisiae* determined by the measurement of the optical density at 600nm is shown of one representative experiment (n>3).

To initiate the analysis of the relation between embryogenesis and ribosome biogenesis we have chosen *A. thaliana* proteins with similarity to the yeast proteins rRNA processing 5 (Rrp5, YMR229C), periodic tryptophan (W) protein 2 (Pwp2, YRC057C), Nin1 (one) binding protein 1 (Nob1, YOR056C), essential nuclear protein 1 (Enp1, YBR247C) and nucleOlar complex associated 4 (Noc4, YPR144C) which are involved in the maturation of the 40S ribosomal subunit (Fig. 1A). Rrp5 is a protein of 190 kDa for which a two domain structure is predicted consisting of twelve tandem S1 RNA binding domains at the N-terminus and six C-terminal tertratricopeptide repeats (TPR) [30]. Yeast Rrp5p acts in the early maturation of ribosomal subunits and its depletion results in the inhibition of the A0, A1, A2, or A3 cleavages [31]–[32]. Pwp2 is also a component of the 90s pre-ribosomal particle in yeast [33]. Depletion of the protein blocks the interaction of the U3 small nucleolar ribonucleoprotein (U3 snoRNP) with the 35S pre-rRNA resulting in an inhibition of the A0-A1-A2 cleavage and leads to a decrease in the level of 18S rRNA and 40S subunits [34]. Nob1 is an endonuclease essential for cleavage at site D [35]–[37]. It contains a PIN (PilT N-terminus) domain that is found in nucleases like yeast Rrp44 [38] or human SMG5/6 [39]. For Enp1 and Noc4, however, only limited information is available [40]. Both proteins are discussed to be involved in the maturation of the 40S subunit. Noc4 is part of the small ribosomal subunit (SSU) processome and a component of the 90S pre-ribosomal particle [41]. A depletion of this protein leads to elevated levels of 35S and 23S (pre)-rRNAs and a reduction in 20S and 27S pre-rRNA [42].

In *Saccharomyces cerevisiae* scNob1 was intensively characterized and the function as an endonuclease was confirmed [35]–[37]. In contrast, no clear function for the other proteins could be identified so far. In localization and pulldown experiments the localization and association with pre-ribosomal complexes was proven for all yeast co-factors and the effect of the depletion of a certain factor on rRNA processing is known. We were interested, if the Arabidopsis (*Arabidopsis thaliana*) orthologs show the same localization and if an effect on rRNA processing in T-DNA insertion mutants can be observed. The aim of this study was to gain first insights into ribosome biogenesis in plants using co-factors participating in the whole 40S maturation pathway. Additionally the influence of a knock-out in ribosome biogenesis related genes on plant development was investigated as former studies suggest a relation between gametophyte and embryo development and functional ribosome maturation [10], [43].

## Materials and Methods

### Plant Material, Growth Conditions and Yeast Complementation

The search for similar *Arabidopsis thaliana* sequences to the yeast sequences was performed with blast [44]. The Arabidopsis sequences with the highest similarity were used to search for T-DNA insertion lines either from Gabi-KAT (GK_834C08, GK_481E08, GK_092G08, GK_053G09, GK_332H07, http://www.gabi-kat.de) or from the Nottingham Arabidopsis Stock Centre (NASC, SA_013032, SA_088516, SA_021098, http://www.arabidopsis.info). As a control the segregated wild-type from the respective T-DNA insertion lines was used. All plants were grown in climate chambers (Percival Scientific Inc.) at a 14 h photoperiod at 120 µmol m^−2^s^−1^ and 21°C at day and 18°C at night. For phenotypic and segregation analysis, seeds were sow out on MS plates containing the selective antibiotic. For yeast complementation *Saccharomyces cerevisiae* BY4741 strains were transformed with vectors for constitutive expression of the proteins used for complementation. Depletion of the yeast protein was induced with Doxycyline at 0 hours to deplete the yeast protein.

### T-DNA Mapping

For verification of the genotype and mapping of the T-DNA position on the genome of mutant plants, genomic DNA from leaves was prepared as described [45]. For higher purity the DNA was treated with RNase A and Proteinase K treatment, extracted with Phenol/Chloroform and precipitated with NaOAc/EtOH. T-DNA insertions were mapped as established [46]. The linker for ligation was generated from a plasmid (pRS415) by digestion with BfaI and PvuII. Ligation products were amplified by nested-PCR using linker-specific and T-DNA left border primers. PCR products were excised from agarose gel, purified (PureLink™ Quick Gel Extraction Kit, Invitrogen™) and sequenced.

### Segregation Analysis

For genotypic analysis seeds of T-DNA insertion lines were sown on MS plates containing the selective antibiotic. After three weeks the plants were transferred to soil. The genotypes of the surviving plants were confirmed by PCR with a T-DNA primer and a left and right border genomic primer. To analyze the transmission of the T-DNA through the male and female gametophyte, flowers of insertion lines were emasculated and pollen from WT was laid on the pistil (female backcrossing). The procedure was repeated *vice versa* for male backcrossing. For each crossing experiment siliques from three independent plants were crossed. The transmission rate was calculated by dividing the number of resistant and sensitive seedlings from one silique. For statistical evaluation of the distribution of transmission rates p-values were determined (Table S4).

### Generation of Transgenic Plant Lines

For the generation of transgenic plant lines, the wild type coding sequence of at*NOB1*was fused with a C-terminal HTP(6xHis-TEV-ZZ)-tag and transformed into the *Agrobacterium tumefaciens* strain GV3101::pMP90 using the freeze-thaw-method [47]. The transfection of wild type Arabidopsis plants with the Agrobacterium strain carrying the fusion construct was carried out via the floral-dip-method as previously described [48]. Because the recommended Silwet L-77 was not available we modified the infiltration medium by adding 0.01% Tween 20. The selection for positive transformed plants was done with Basta® (Bayer CrobScience). A positive expression of the construct in the selected plant lines was verified by western blot analysis using the goat anti-rabbit IgG peroxidase antibody (αHTP, Sigma Aldrich). For analysis of expression, co-suppression and rRNA processing the T3 generation after transformation was used.

### RNA Isolation and Northern Blotting

RNA was isolated using the NUCLEOSPIN® RNA II kit (Macherey-Nagel). RNA from seeds, siliques and roots was isolated as described [49] and further purified by NUCLEOSPIN® RNA II columns. Northern transfer and hybridization were performed as described [50]. Hybridization probes are listed in Table S3.

### cDNA Synthesis and Quantitative RT-PCR Analysis

First-strand cDNA was synthesized using the M-MuLV reverse transcriptase (Fermentas) following the manufacturers protocol. An oligo-dT primer (Table S2) was used for reverse transcription. For the determination of the relative expression level of all investigated genes in wild-type and mutant plants RNA from leaves of three individual plants was isolated. For each primer pairs standard curves were made to determine the optimal cDNA dilution. For each cDNA synthesis 1 µg of RNA was reverse transcribed. The cDNA was diluted 1∶6 before quantification of transcript levels. The qRT-PCR was performed as described [51]. The Ct values of the genes of interest were normalized to the expression level of at*ACT2* and the ratios were calculated using the formula: relative expression level 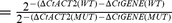. For the relative expression level of the investigated genes in different developmental stages and tissues three biological replicates were used. The reverse transcription was done using 400 ng of RNA for each sample. The cDNA again was diluted 1∶6 and the qRT-PCR was done as described [51]. The Ct values of the genes of interest were normalized to the expression level of at*UBI3* and the relative expression level was calculated using the formula: relative expression level 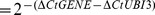.

### Antibody Generation

Peptide antibodies against atRrp5 and atPwp2 were generated by immunization of guinea pigs with two peptides for each protein (PSL, Heidelberg). Antibodies were purified by incubation of the sera with peptide coupled iodoacetyl-activated agarose beads (SulfoLink Columns, Thermo Scientific) following the manufacturers protocol (PSL, Heidelberg). The coding sequence of atNob1 and atEnp1 were cloned into pQE80 (Qiagen) to create N-Terminal 21xHis-TEV fusion construct for expression in *E.coli* BL21 Star pRosetta at 18°C for 16–20 h after induction with 0.25 mM IPTG. After purified over NiNTA (Qiagen) the proteins were used for antibody generation by immunization of rabbits (Dr. Pineda, Berlin). For antibody purification from serum the proteins were further cleaved by GST-TEV protease and purified over Glutathion-Sepharose and a sephacryl S-200 column before coupling to activated CNBr-Sepharose (GE Healthcare) according to the manufacturers protocol. Serum was incubated with the matrix and specific antibodies eluted using 0.2 M glycine pH 2.2, neutralized and precipitated with saturated ammonium sulfate.

### Light and Fluorescence Microscopy

To visualize the embryo development, seeds were dissected from siliques and bleached in Hoyers’ solution [52] for 3 h or overnight (Olympus CKX41). For GFP-fluorescence measurements, the coding sequences of atNob1, atNoc4 and atEnp1 were cloned into the pRT-vector to generate C-terminal GFP fusions under control of a double 35S promoter. As a nucleolar localization control at*FIB2* (At4g25630) was cloned in front of mCherry into the same vector and co-transformed with the GFP-fusion constructs. Arabidopsis leaf mesophyll protoplasts were isolated, transformed and visualized as described [53]. To analyze the localization of atPwp2 and atRrp5 indirect immunofluorescence in Arabidopsis root tips was performed as described [54]. Briefly, three to five day old seedlings were fixed with 4% paraformaldehyde, laid on SuperFrost®Plus glass slides (VWR) and digested with driselase (Sigma). The root tips were blocked with 3% BSA and incubated with the primary antibody overnight at 4°C. Primary antibodies against Fib (Fibrillarin monoclonal antibody 38F3, Thermo Scientific), atRrp5 and atPwp2 were diluted 1∶50. Secondary Cy2-conjugated antibody (goat α-mouse or α-guinnea pig IgM-Cy2, Dianova) was diluted 1∶500. The fluorescence was visualized by CLSM with a TCS SP5 (Leica).

### Sucrose Gradients and rRNA Cleavage Assay

For sucrose density centrifugation cell extract from Arabidopsis cell culture [55] was prepared by grinding in liquid nitrogen followed by resuspension in 5 volumes of extraction buffer (50 mM Tris pH 7.5, 100 mM NaCl, 5 mM MgCl_2_, 1 mM DTT, 1% NP-40 supplemented with 10 mM ribonucleoside-vanadyl complex [NEB] and 1% plant protease inhibitor cocktail from Sigma). Centrifugation was carried out as described [51]. Fractions were precipitated with MetOH/Chloroform and subjected to SDS-PAGE and Western Blotting with indicated antibodies. RNA was isolated as described [51] and mature rRNAs analyzed in 8% polyacrylamide/8 M Urea after ethidium bromide (EtBr) staining. The Nob1 cleavage assays were performed as described [56].

## Results

### Ribosome Biogenesis Co-factors are Conserved in Plants

We have selected five proteins from yeast which cover the whole 40S biogenesis pathway and for which an association with pre-ribosomal subunits was experimentally confirmed e.g. by pull-down analysis. We have searched for orthologs in *Arabidopsis thaliana* and named the identified factors according to the yeast standard name. For all five factors only one homolog was identified in the genome of *A. thaliana,* namely atRrp5 (At3g11964), atPwp2 (At1g15440), atNob1 (At5g41190), atEnp1 (At1g31660) and atNoc4 (At2g17250). The selected factors are generally conserved between mammals, fungi and plants (Fig. 1B), and Nob1 is even present in archaea [56]. The similarity of the plant proteins to their fungal counterparts varies. While Pwp2, Rrp5 and Enp1 exhibit the highest conservation, the similarity is lowest for Nob1 (Fig. 1B, Fig. S1). To test the functional conservation of the *Arabidopsis* proteins we analyzed the growth of yeast depletion strains expressing either the yeast protein or the respective *Arabidopsis* homolog (Fig. 1C). Unexpectedly from the conservation profile, only using atNob1 a partial complementation was observed, while the other *Arabidopsis* proteins did not complement the yeast depletion phenotype. The growth analysis of the Rrp5 depletion strain expressing atRrp5 could not be carried out due to the size of the *RRP5* gene. The expression of the *Arabidopsis* proteins atEnp1 and atNob1 was verified by western blot analysis (Fig. S2). However, based on sequence similarity we assume that the selected plant factors are also involved in ribosome biogenesis although the *Arabidopsis* proteins could not complement yeast depletion phenotypes.

### The Putative Ribosome Biogenesis Co-factors Show Distinct Expression Patterns

**Figure 2:**
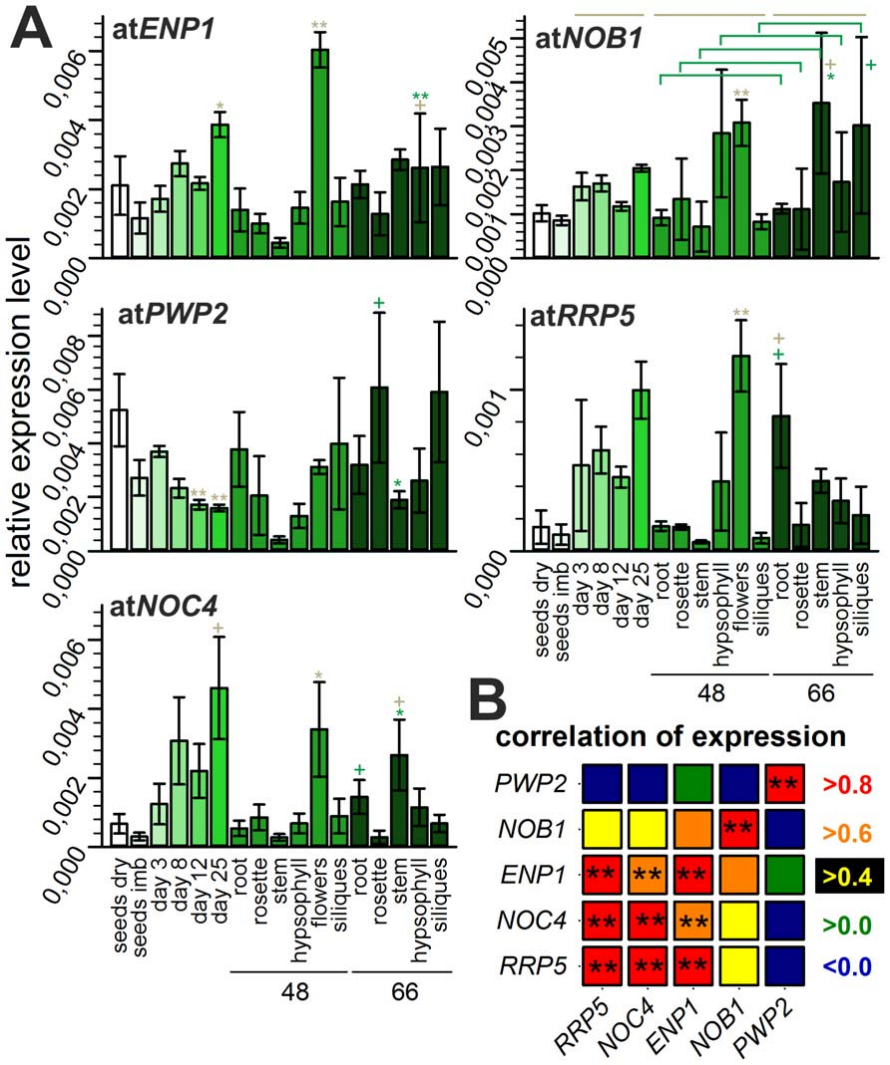
Developmental stage and tissue dependent mRNA abundance. A, Relative expression levels of the mRNAs are depicted in different shades of green. Error bars illustrate standard deviation of at least three independent results. The age of the investigated tissues is indicated with 48 and 66 (days) on the x-axis below the corresponding samples. The significance of changes was determined by a two-tailed paired Student’s t-test for developmental stages from day 8 to 25 in comparison to day 3 and for different tissues at day 48 or 66 normalized to values for rosette (indicated by grey lines and grey asterisks or plus). In addition, the change of expression in a specific tissue between day 48 and day 66 was analyzed (green brackets, green asterisk or plus). A plus indicates p-values below 0.005, one asterisk indicates p-values below α = 0.001 and two asterisk a p-value below α = 0.0001 B, The correlation of the expression profiles of the investigated factors. The color indicates the correlation factor and the two asterisks again a p-value below α = 0.0001. The p-values are related to the correlations and roughly indicates the probability of an uncorrelated system producing datasets that have a Pearson correlation at least as extreme as the one computed from these datasets.

We determined the relative expression levels of the genes coding for the selected factors in different tissues and at different developmental stages of *A. thaliana* by quantitative RT-PCR (qRT-PCR, Fig. 2A). The transcripts of all genes are present in all investigated stages (Fig. 2A), but differences between the expression patterns of the different genes exist. For *ENP1*, *NOB1*, *RRP5* and *NOC4* a higher expression in flowers compared to all other tissues was observed. While expression of *ENP1* and *NOB1* is comparable in all developmental stages and tissues (apart from flowers) analyzed, the expression of *RRP5* and *NOC4* was also higher in 8 to 25 day old plants (Fig. 2A). Thus, the correlation of the expression patterns was the highest between *NOC4* and *RRP5* (Fig. 2B). The expression pattern of *PWP2* showed the lowest correlation to all other ribosome biogenesis co-factors, as the expression of *PWP2* was equal in all investigated developmental stages and tissues.

As the expression of the reference gene *UBIQUITIN 3* (*UBI3*) is not equal in all probes tested (Fig. S3A), we also evaluated the expression pattern and correlation with normalization to amounts of RNA used for reverse transcription (see methods part). Again a high expression in flowers and young plants was observed and the correlation profile determined did not change drastically, e.g. the expression pattern of *PWP2* shows the least correlation to the expression of other factors (Fig. S3B). We further compared the expression pattern based on data deposited in genevestigator (https://www.genevestigator.com/gv/plant.jsp). Consistent with the qRT-PCR data presented in here, the highest expression was reported in reproductive and strongly dividing tissues (flowers, roots, cell culture; Fig. S4).

In summary, the genes investigated are predominantly expressed in reproductive or strongly dividing tissue. This result is consistent with previous findings, where genes related to ribosome biogenesis are highly expressed in tissues with a high demand on ribosomes [43], [57].

### The Localization of the Putative Ribosome Biogenesis Co-factors in Arabidopsis

**Figure 3:**
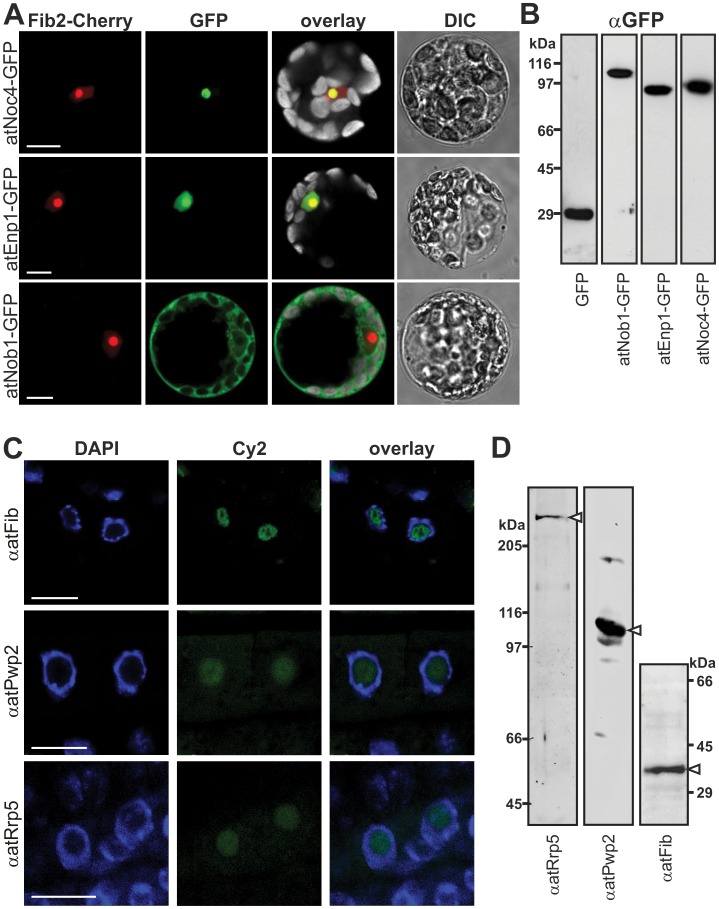
Cellular localization of ribosome biogenesis co-factors. A, Arabidopsis mesophyll protoplasts were co-transformed with C-terminal GFP fusion constructs indicated (left) and atFib2-mCherry (nucleolar marker). Cherry- (red), GFP- (green), chlorophyll auto-fluorescence (grey, in overlay) and DIC image is shown. Scale bar = 10 µm. B, Arabidopsis mesophyll protoplasts transformed with C-terminal GFP fusion constructs were lysed, subjected to SDS-PAGE and immunodecorated with GFP. C, Arabidopsis root tip cells were incubated with primary antibodies (left) and secondary antibody labeled with Cy2 fluorophore (green). Tissues were stained with DAPI (blue) to visualize the nucleus. Scale bar: 10 µm. D, Arabidopsis cell culture extract subjected to SDS-PAGE followed by Western Blot analysis using the indicated antibodies. White arrows point to expected migration of the protein.

Having established the expression of the identified genes, we analyzed the cellular localization of the encoded proteins. We generated expression constructs of full length atNoc4, atEnp1 and atNob1 as C-terminal GFP fusions (Fig. 3A), which were expressed in *A. thaliana* protoplasts (Fig. 3B). In addition, we co-transformed the protoplasts with a *FIBRILLARIN2* (*Fib2*)-mCherry construct (Fig. 3A, F-Cherry) as nucleolar marker. Consistent with the assignment to the ribosome biogenesis pathway based on the yeast homologue (Fig. 1) we observed exclusively nucleolar localization for atNoc4 (Fig. 3A) and nucleolar and nucleoplasmic localization for atEnp1. For atNob1 most of the GFP fluorescence was observed in the cytoplasm and only a minor signal was detected in the nucleoplasm (Fig. 3A). This is consistent with the function of scNob1, which cleaves 20S pre-rRNA in the cytoplasm.

Unfortunately, atPwp2 and atRrp5 transiently expressed in protoplasts were degraded (atPwp2) or not expressed (atRrp5). Thus, we generated peptide antibodies against these proteins, and analyzed the protein localization in root tissues by immunofluorescence (Fig. 3C). The specificity of the peptide antibodies was tested by western blot analysis using *A. thaliana* cell culture extracts (Fig. 3D). We observed an exclusively nucleolar signal for the Rrp5 and Pwp2 antibody in *Arabidopsis* roots (Fig. 3C). The localization of the nucleus within the cell was visualized with DAPI staining. To verify the localization of the nucleolus we used a Fib antibody (Fig. 3D). The fluorescence signal could be clearly localized to the nucleolus because this structure shows a weak DAPI stain and the characteristic central cavity termed “nucleolar vacuole” is observed [58]. Unfortunately, the antibodies against atNob1 and atEnp1 raised from recombinant protein were not suitable for immunofluorescence in *Arabidopsis* roots. Nevertheless, the observed localization by fusion protein analysis or immunofluorescence of the plant proteins is in agreement with the localization of the five ribosome biogenesis co-factors in yeast.

### AtNob1 is an Endonuclease Cleaving Pre-rRNA at Site D

**Figure 4:**
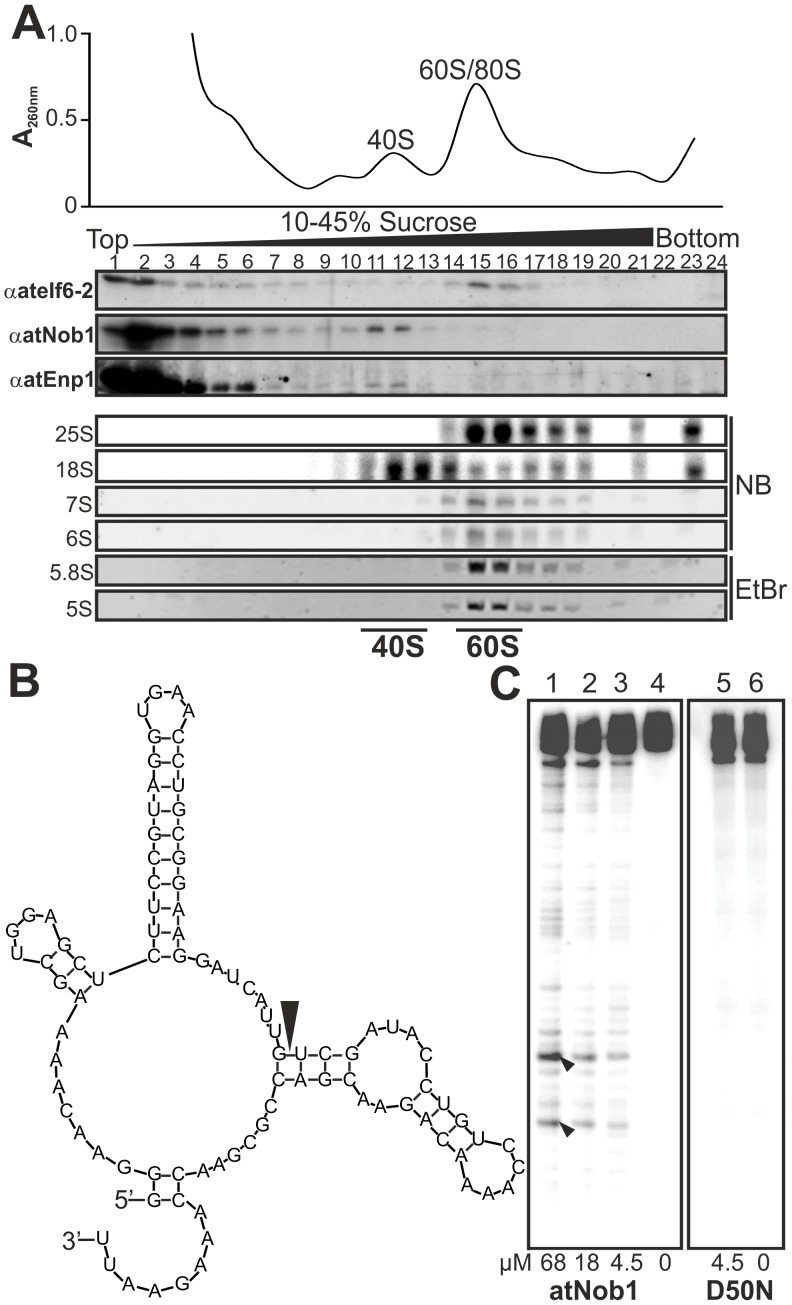
AtNob1 and AtENP1 are components of the 40S pre-ribosome. A, Arabidopsis cell culture lysate was applied to continuous sucrose gradient centrifugation. The absorption profile is shown on top. Fractions were collected and subjected to SDS-PAGE and Western blot analysis with indicated antibodies. RNA of the fractions was isolated and rRNA content was determined by northern blot analysis (NB) or EtBr staining. B, Secondary structure prediction of the RNA probe used for the cleavage assay is shown. The black arrow points to the predicted cleavage site D. C, Internally labeled *in vitro* transcribed RNA was incubated with recombinant atNob1 and the D50N mutant. Black arrows indicate the expected cleavage products.

For atNob1 we mainly observed cytoplasmic localization (Fig. 3), although in yeast Nob1p associates with pre-ribosomal complexes already in the nucleolus [59]. To support a functional relation of atNob1 to ribosome biogenesis we lysed *A. thaliana* cell culture cells and fractionated pre-ribosomal complexes by sucrose density centrifugation (Fig. 4A). The fractions were collected and the migration of atNob1 was visualized by western blot analysis. Additionally, the migration of atEnp1 was tested. The specificity of the generated antibodies was verified prior to the experiment (Fig. S5). For the remaining proteins, atRrp5, atPwp2 and atNoc4, no association with pre-ribosomal complexes could be analyzed, because the available peptide antibodies were either not affine enough to detect low amounts of protein in the gradient fractions (Rrp5, Pwp2) or no antibody for the protein was at hand (Noc4). Additionally, the verification of the 90S or 40S co-migration by immunodecoration for atPwp2, atRrp5 and atNoc4 was difficult because we were not able to extract all early pre-ribosomal particles by lysis of the nucleolus. For the cytoplasmic atNob1 and the nuclear atEnp1 high amounts were found in the top fractions of the gradient (Fig. 4A, fraction 1–4), representing non-associated factors. However, both proteins were also enriched in fraction 11 and 12, which contain (pre-)40S subunits as judged from the absorption profile and the presence of the mature 18S (Fig. 4A, lower panel). Consistently, ateIf6-2 which is associated with the pre-60S subunit [60] was observed in fractions 14–17 representing the 60S complexes based on the absorption profile (Fig. 4A). The migration of the pre- and mature 60S particle was further verified by the detection of 7S/6S and 25S/5S/5.8S in northern blot analysis (Fig. 4A, lower panel). The major pre-rRNAs for pre-40S (20S or P-A3)) and pre-60S (27S) were also detected in fractions 11/12 and 14–17, but both showed strong degradation.

Nob1 is involved in the D-cleavage of the rRNA in yeast and archeae and thus we tested whether the recombinantly produced atNob1 can catalyze the same process. A RNA fragment containing the sequence of the D cleavage site of the *A. thaliana* rRNA (Fig. 4B) was radiolabeled and incubated with purified recombinant atNob1 (Fig. 4C). We observed a protein concentration dependent accumulation of the two expected rRNA cleavage products (lane 1–3). These products were specific for functional atNob1, because atNob1 with the exchange of the essential aspartic acid [37], *atNob1-D50N*, did not possess the catalytic activity (lane 5). Unfortunately, the yield for the recombinant mutant of Nob1 was very low that a maximal concentration of 4.5 µM could be tested. However, the same concentration of wild-type protein yielded a clear cleavage product, while the mutant Nob1 shows no activity. Thus, the association with the pre-ribosomal complexes and the observed activity of Nob1 supports a function of atNob1 as endonuclease involved in 40S maturation. For atEnp1 an 40S association was also observed suggesting an involvement in ribosome biogenesis as well. For all other plant proteins the localization gives a hint to a participation in ribosomal subunit assembly but an association with pre-ribosomal subunits could not be experimentally confirmed.

### The Putative Ribosome Biogenesis Co-factors are Essential in Arabidopsis

**Figure 5:**
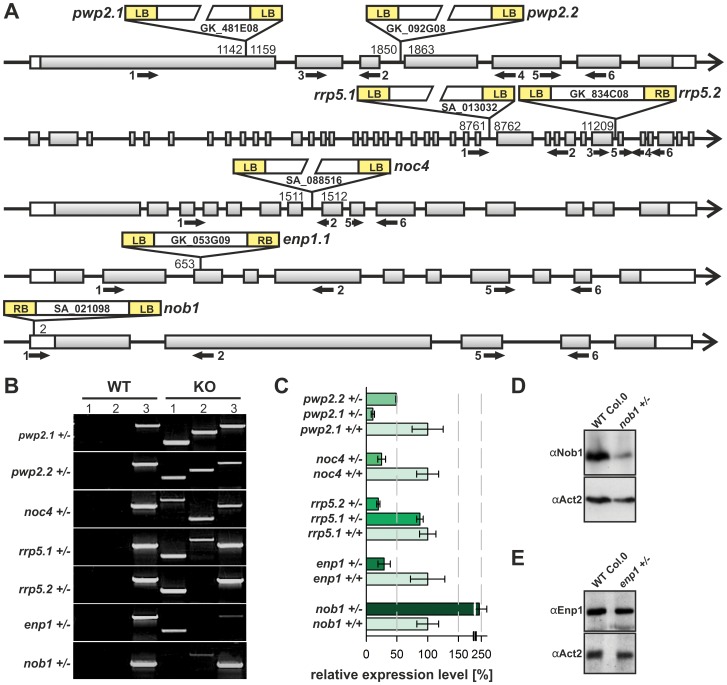
Analysis of T-DNA insertion mutants. A, Positions of the T-DNA within the genes are shown. Accession numbers of the plant lines and the name used here is given. The base position verified by T-DNA mapping (Table S1) is indicated on the left or right border of the insertion. Black arrows indicate primer binding sites used for the analysis (Table S2). B, Segregation state of insertion lines was verified with PCR. The T-DNA left border primer was combined either with the forward (lane 1) or reverse genomic primer (lane 2). For lane 3 the forward and reverse genomic primers were used. C, mRNA-levels in wild-type and mutants were analyzed by qRT-PCR. Values were normalized to *ACT2* and the wild-type level was set to 100% for comparison to the expression level in mutants. Oligonucleotides are listed in Table S2. D, Protein levels of atNob1 in WT and *nob1+/−* were determined by immunodecoration of plant extract with αNOB1 or αACT2 antibodies (loading control). E, Protein levels of atEnp1 in WT and *enp1+/−* were determined by immunodecoration of plant extract with αEnp1 or αAct2 antibodies (loading control).

The functional relevance of the factors was subsequently explored by analyzing corresponding T-DNA insertion lines (Fig. 5A). Two T-DNA insertion lines were available for at*RRP5*, at*PWP2* and at*ENP1* (*rrp5.1,* SA_013032; *rrp5.2,* GK_834C08; *pwp2.1,* GK_481E08; *pwp2.2,* GK_092G08; *enp1.1,* GK_053G09; *enp1.2,* GK_332H07). Unfortunately, only one suitable T-DNA insertion line exists for the other two factors (*noc4,* SA_088516; *nob1,* SA_021098).

We confirm the position of the T-DNA given by the border sequences deposited in the databases by genomic mapping [46]. The border sequences of the T-DNAs were determined by sequencing of PCR products which contain the left border genomic sequence (Table S1). For all GABI-Kat lines we found the same base pair position as provided by the stock center (Fig. 5A), while SALK T-DNA insertion borders are distinct of those deposited in the database (Table S1). For four of the seven lines we observed a back-to-back insertion of two T-DNAs (Fig. 5A). Further, with the exception of the *enp1.2* line we did not observe additional T-DNA insertions in the genome of the different lines. For *enp1.2* we observed a second position of T-DNA insertion. To isolate plants with single T-DNA insertion, *enp1.2* line was backcrossed with wild-type. We have screened approximately 120 plants of the second generation after backcrossing, but we were unable to separate the two insertions. Thus, we excluded this line from further analysis.

All lines revealed a heterozygous status based on the amplification of the wild-type gene (Fig. 5B, lane KO, 3). To justify the use of the different insertion lines we determined the transcript level in heterozygous plants by qRT-PCR with primers amplifying a 100 bp fragment from the 3′ end of the coding sequence. The primers cover an exon-exon boundary to exclude genomic DNA contamination (Fig. 5C). For *rrp5.2+/−, pwp2.2+/−, noc4*+/− and *enp1*+/− we observed the expected transcript reduction of around 50% as compared to wild-type. The reduction in expression was even more drastic in *pwp2.1+/−* where only 10% of the transcript could be detected. In contrast, the transcript level of at*RRP5* in *rrp5.1+/−* plants was only slightly reduced when compared to wild-type. To our surprise, the transcript abundance of *NOB1* was about 2.5 fold higher in the *nob1+/−* plants when compared to wild-type (Fig. 5C). However, this transcript enrichment does not lead to a functional protein, as the Nob1 protein is significantly reduced in the heterozygous mutant plants as determined by western blotting (Fig. 5D). Therefore, we conclude that the enhanced transcript level does not account for an increase in protein content.

We also analyzed the protein level in *enp1+/−*, which was not significantly reduced in total plant extracts of 14 day old plants in comparison to wild-type (Fig. 5E). For atNoc4, atRrp5 and atPwp2 the protein level could not be determined in the heterozygous T-DNA insertion lines. On the one hand, we were unable to generate a suitable antibody against atNoc4. On the other Hand, the expression of atRrp5 and atPwp2 was very low or the peptide antibodies against these two proteins not affine enough for denatured protein to determine differences in protein levels in wild type and mutant plants. Nevertheless, alike the yeast proteins atRrp5, atPwp2, atNoc4, atEnp1 and atNob1 are essential and the lethality of the homozygous knockout indicates a high importance of the protein in an essential pathway.

### The Heterozygous T-DNA Insertion Mutants Show Alterations in rRNA Processing

**Figure 6:**
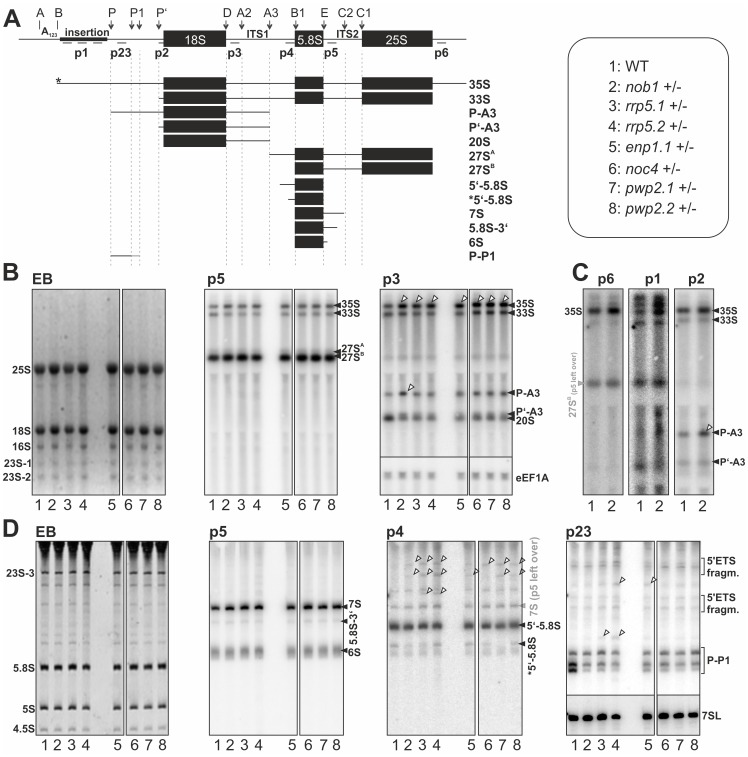
rRNA processing in wild-type and mutant plants. A, The scheme of pre-rRNA processing indicating cleavage sites (top) and the expected intermediates is shown. Names of intermediates (right) and numbers used in B–D (left) are given. Priming sites for Northern probes are indicated. Stars indicate unknown processing positions. B, RNA from flowers indicated (bottom) was separated on agarose gel, stained with EtBr (left) for visualization of mature rRNAs or Northern blotted with probe p5 (middle) or p3 (left) to detect pre-rRNA. The eEF1A RNA was probed as control (see right). Migration of rRNA intermediates is indicated (right). C, RNA from wild-type and *nob1*+/− plants was probed with p6 (left), p1 (middle) or p2 (left). Migration of rRNA intermediates is indicated (right). D, RNA from plants indicated (bottom) was separated by acrylamide gel, stained with EtBr (left) for visualization of mature rRNAs or Northern blotted with probe p5, p4 or p23 to detect pre-rRNA. Migration of rRNA intermediates is indicated (right). The 7SL RNA was probed as control; shown on the right. For B–D: alterations between wild-type and mutant lines are indicated by tilted arrows. Please note, all probes were used on the same blot and images were processed simultaneously.

The heterozygous lines did not show significant differences with respect to developmental rate, growth, plant size or flowering in comparison to wild-type (Fig. S6). Next, we analyzed the molecular properties of these plants such as the pre-rRNA processing pattern (Fig. 6A). RNA isolated from wild-type or mutant flowers were separated by agarose (Fig. 6B,C) or acrylamide gel electrophoresis (Fig. 6D). The EtBr-stain visualizes the most abundant mature cytoplasmic rRNAs 25S and 18S (6B) and 5S and 5.8S (Fig. 6D). Apart from these, the chloroplastic rRNA 16S and 23S are also detectable (Fig. 6B,D). The 23S rRNA is further processed at so called “hidden breaks” [61], which leads to a fragmentation in three different 23S products (23S-1, 23S-2, 23S-3; Fig. 6B,D). The analysis of the pre-rRNA transcripts in wild-type revealed a distribution comparable to previous reports [8]. We detected two large abundant pre-rRNA species assigned as 35S and 33S (Fig. 6A–C). Furthermore, we were able to detect the equivalent rRNA precursors as already reported for yeast. Also in *Arabidopsis* the 27SA and 27SB are present (Fig. 6B, p5). For the smaller precursors also associated with the large pre-ribosomal subunit we could assign 7S, 6S and 5′ and 3′ extended 5.8S rRNAs to the pathway in *Arabidopsis* (Fig. 6D, p4, p5). The major difference to yeast was observed in the processing of 18S rRNA precursors in *Arabidopsis*. In yeast the first processing step involves cleavage at A2 which produces the 20S pre-rRNA. The 20S pre-rRNA in Arabidopsis is an elongated transcript ending at cleavage site A3 [8]. This 20S- precursor is detectable in *Arabidopsis* (Fig. 6B, p3), but in contrast to yeast the first cleavage in *Arabidopsis* takes place at A3, resulting in the formation of the P-A3 fragment (Fig. 6B, p3). This fragment is the equivalent to 23S in yeast, which is an aberrant processing product, when cleavage at A2 is aborted [42]. Thus, P-A3 in *Arabidopsis* can also be called 23S-like rRNA. Additionally a third 18S precursor, the P’-A3 Fragment, is present in *Arabidopsis* (Fig. 6B, p3).

The comparison of the rRNA processing in wild-type and T-DNA insertion mutants revealed some significant alterations. For all lines an accumulation of 35S above the 33S rRNA was observed (Fig. 6B). In the heterozygous lines of the three factors acting early in ribosome biogenesis (Noc4, Rrp5, Pwp2) the maturation of 5.8S rRNA was affected, especially the processing at the 5′ end (Fig. 6D, p4). For all plant lines investigated the level of the 27S rRNA (Fig. 6B, p5) and the major 5.8S precursors 7S and 6S were not changed (Fig. 6D, p5). The most significant alteration were observed for *nob1+/−* plants (Fig. 6B,C). Although, an enrichment of P-A3 was also observed for other plants (enp1+/−, *pwp2*+/−, *noc4*+/−), the accumulation of the 23S-like precursor P-A3 was most prominent in *nob1*+/− (Fig. 6B,C; Fig. S7). However, as expected from the heterozygosity of the plants and the importance of functional ribosomes in general, all rRNA intermediates were observed in all plant lines and the loss of one particular precursor or mature rRNA could not be observed.

**Figure 7:**
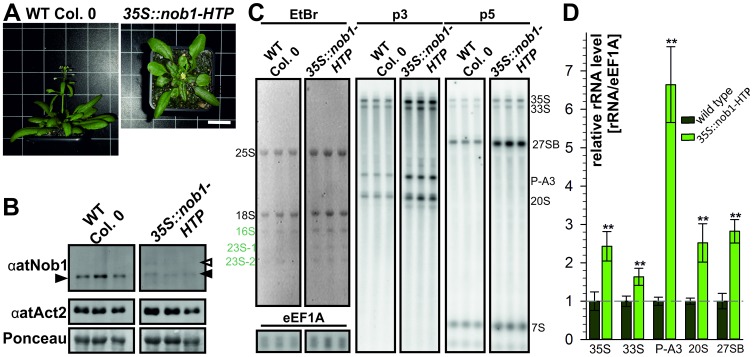
Analysis of co-suppression mutants of *nob1*. A, To visualize the growth and flowering phenotype of wild type and *nob1* co-suppression mutants two representative 30 day old plants are shown. Scale bar: 30 mm for both panels. B, For protein expression study of wild type and *nob1* co-suppression mutants (upper panel) three independent wild type plants and three independent *nob1* co-suppression mutants (independent transformation events) were used. As loading control αActin antibody (middle panel) and Ponceau staining (lower panel) are depicted. C, For northern blot analysis total RNA from leaves was loaded on a agarose gel. Three representative, independent plant lines are presented. EtBr staining of the gel is shown (left). As a loading control a probe against eEF1A was used. The migration of the pre-rRNAs is indicated on the right. D, The northern blot quantification of the major transcripts (35S, 33S, P-A3, 20S and 27SB) was normalized to the signal of eEF1A after background correction. For wild type three replicates were used. For the co-suppression mutants of *nob1* six plant lines derived from four independent transformation events were used for quantification. To statistically analyze the changes of pre-rRNA values a Students’s t-test was performed. The two asterisk indicate a p-value below 0.005.

To verify the effects shown for *nob1*+/− we generated co-suppression plant lines (Fig. 7A). For these plants the protein level of the endogenous protein is significantly reduced due to high expression of a 35S driven nob1-HTP transcript (Fig. 7B). The drastically reduced Nop1 protein level led to alterations in leaf morphology (Fig. 7A) and to inhibition of inflorescence elongation and thus, a loss of reproduction. We analyzed the rRNA processing in this plant lines (Fig. 7C). We observed a strong accumulation of the P-A3 by more than 5 fold (Fig. 7D), as well as a moderate increase of the 35S/33S precursors and the 20S pre-rRNAs (Fig. 7D). We also tried to investigate co-suppression lines for the other ribosome biogenesis co-factors, but no plants survived to a developmental stage, were a reasonable molecular or biochemical analysis would have been possible.

In summary, we could clearly show the involvement of the ribosome biogenesis co-factors in this pathway, as defects in rRNA processing already occur in the heterozygous state. Especially the involvement of atNob1 in the processing of the 18S rRNA precursors was shown in heterozygous and co-suppression plant.

### Arabidopsis Mutants Show Defects in Embryo and Gametophyte Development

**Figure 8:**
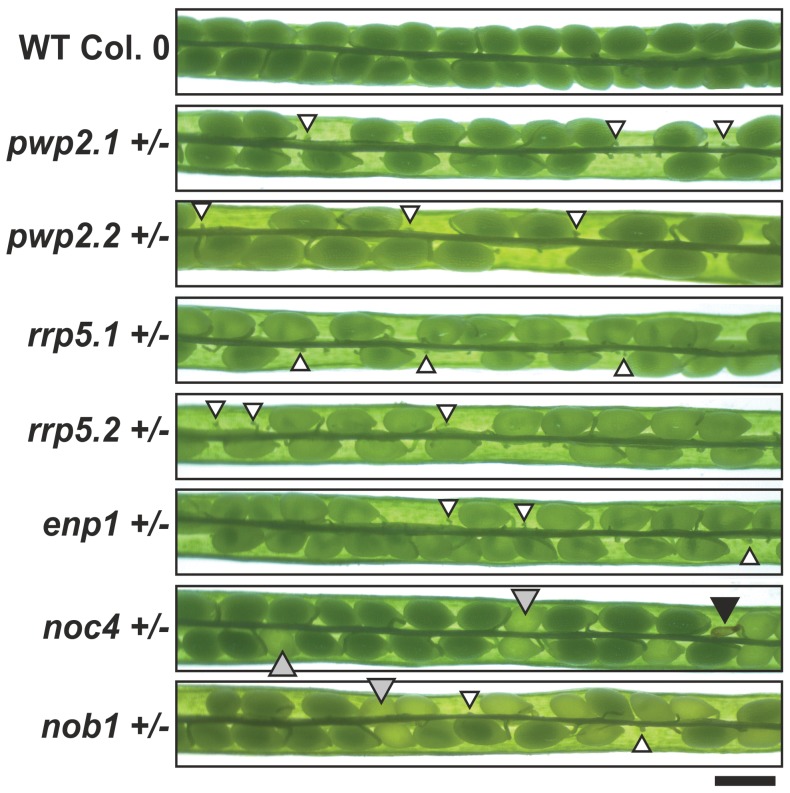
Embryo development of heterozygous mutants. Wild-type and indicated mutants siliques were dissected to visualize the seeds. Black arrows indicate aborted seeds, white arrows undeveloped seeds and grey arrows pale seeds. Scale bar: 200 µm.

**Figure 9:**
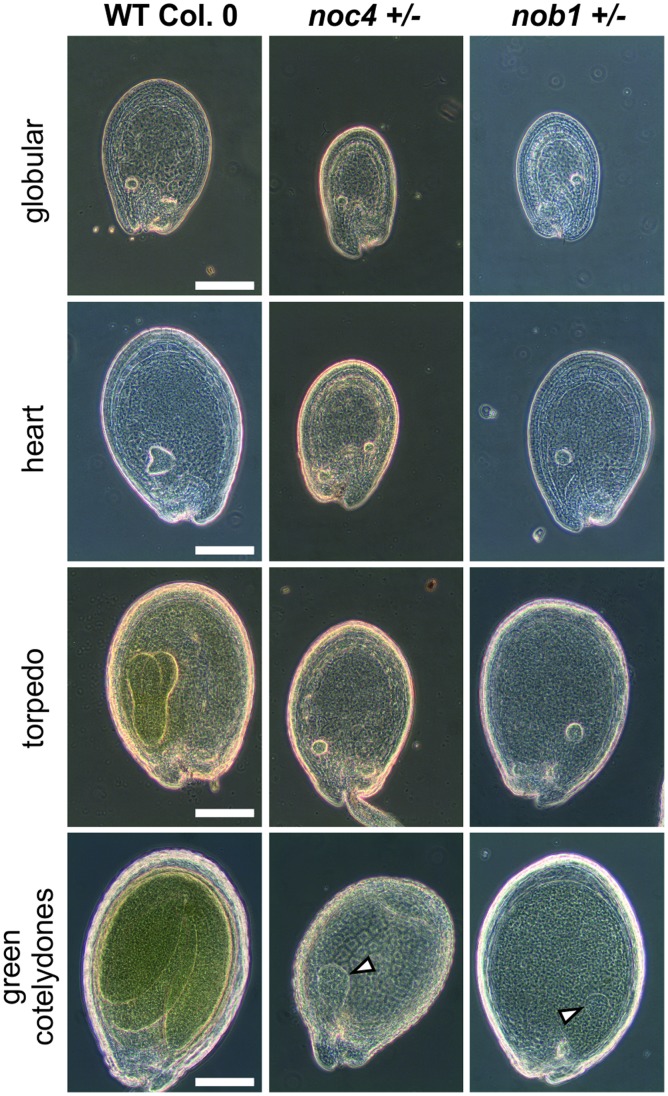
Embryos in pale seeds of *noc4+/−* and *nob1+/−* arrest in globular stage. Seeds from wild-type and insertion lines were bleached for visualization with a phase contrast microscope. Scale bars: 100 µm.

Although the mutant plants do not show strong changes in the overall morphology, we observed significant alterations in the size of siliques, which is reduced by at least 25% for the heterozygous mutant lines of *pwp2, rrp5* and *enp1* (Table 1, Fig. S8) when compared to wild-type. Analysis of the seed content of siliques from these lines revealed the presence of small non-developed or early aborted seeds (Fig. 8) leading to an overall seed reduction of around 50% (Table 1). The siliques of *noc4+/−* or *nob1+/−* were not as drastically reduced in size (Table 1) and seed abortion was only observed for the latter one (Fig. 8). As a consequence, the number of seeds per silique of *nob1+/−* plants was reduced by 15%, while it was comparable to wild-type for *noc4+/−* plants. However, for both lines we observed around 25% pale seeds (Table 1) which lead to the reduction of the germination rate of these two lines (Table 1). Bleaching of the pale seeds in Hoyer’s solution showed an arrest of the embryo development in the globular stage for seeds of *nob1+/−* and *noc4*+/− (Fig. 9). In contrast to wild type where the embryo passes through all developmental stages up to the final green cotelydone stage, the embryo in the pale *noc4+/−* or *nob1+/−* seeds is unable to initiate asymmetric cell divisions to form the heart stage. As a consequence the embryo only slightly increases in size but no mature embryo is formed (Fig. 9, bottom panel).

**Table 1:** Seed set and germination rate of heterozygous mutants.

mutant line	Silique length [%]^1^	seeds per silique [%]^2^	no. of pale seeds [%]^3^	germination rate [%]^4^
***pwp2.1+*** ***/−***	74±5	51±10	−	100
***pwp2.2+*** ***/−***	76±5	57±8	−	100
***rrp5.1+*** ***/−***	74±5	56±7	−	100
***rrp5.2+*** ***/−***	75±4	58±8	−	100
***noc4+*** ***/−***	92±5	100±11	20±5	82±5
***enp1.1+*** ***/−***	72±7	49±8	−	100
***nob1+*** ***/−***	82±5	84±9	25±6	93±2

Seeds were sowed out on selection plates containing kanamycin for Salk lines or sulfadiazine for Gabi lines. The plants were grown under long day condition for 3 weeks.

1 From 3 independent plants 15 siliques each were measured. The length was calculated according to the length of wild-type siliques, which were set to 100%.

2 Percentage was calculated according to wild-type seed set, which was set to 100%.

3 Percentage was calculated according to the amount of seeds in the heterozygous silique.

4 Percentage was calculated according to wild-type, which was set to 100%.

To clarify the transmission of the T-DNA through male and female gametophyte, we quantified the rates by selfing, male or female backcrossing. For Mendelian segregation the transmission rates should be 2∶1:1 for selfing:male backcrossing:female backcrossing [28], while the segregation changes to 1∶0:1 or 1∶1:0 when gametophyte affecting mutations occur. However, it has been manifold described and discussed that experimentally determined values of transmission rates (TRs) are typically lower than the ideal assumption [62], [63]. We observed clear differences between determined TR of the analyzed lines (Table 2). Comparing the probabilities for Mendelian and non-Mendelian behavior the conclusions on these distributions are found to be statistically significant (Table S4) although we realized large standard deviations for the results.

**Table 2:** Transmission rates of selfing and backcrossed mutants.

mutant line	backcrossing^1^	germinated seeds	resistant seedlings	sensitive seedlings	transmission rate [AB^R^/AB^S^]
***pwp2.1+*** ***/−***	Selfing	342	131	211	0.63±0.15
	Paternal	122	49	73	0.66±0.19
	Maternal	109	0	109	0.00±0.00
***pwp2.2+*** ***/−***	Selfing	387	156	231	0.56±0.23
	Paternal	150	75	75	0.88±0.23
	Maternal	280	3	277	0.01±0.02
***rrp5.1+*** ***/−***	Selfing	332	142	190	0.80±0.24
	Paternal	541	235	306	0.82±0.22
	Maternal	115	4	111	0.02±0.03
***rrp5.2+*** ***/−***	Selfing	447	124	323	0.40±0.17
	Paternal	855	340	515	0.72±0.25
	Maternal	272	0	272	0.00±0.00
***noc4+*** ***/−***	selfing	428	281	147	1.95±0.32
	paternal	521	258	263	1.01±0.16
	maternal	682	344	338	1.03±0.20
***enp1.1+*** ***/−***	selfing	179	6	173	0.03±0.02
	paternal	193	3	190	0.01±0.03
	maternal	176	0	176	0.00±0.00
***nob1+*** ***/−***	selfing	325	163	171	1.00±0.34
	paternal	418	101	308	0.35±0.16
	maternal	336	26	317	0.07±0.10

Seeds were sowed out on selection plates containing kanamycin for Salk lines or sulfadiazine for Gabi lines. The plants were grown in long day condition for 3 weeks.

1 Paternal backcrossing was performed with the insertion mutant as pollen donor. For maternal backcrossing flowers of mutant lines were fertilized with wild type pollen. For each heterozygous insertion line 10–30 flowers were backcrossed. The term selfing describes flowers that were self pollinated.

For *pwp2* or *rrp5* mutants we observed a transmission rate between 0.4 and 0.8 for selfing. For the paternal backcrossing we observed a somewhat higher TR compared to selfing (Table 2) suggesting that transmission through the male gametophyte was not affected. In contrast, maternal backcrossing revealed a TR of zero documenting that a female gametophyte defect exists for these two factors (Table 2, Table S4). In case of *noc4+/−* where no seed set reduction was observed we observed a TR of 1.95 for selfing, which shows that the mutation can be maternally and paternally inherited and follows a classical Mendelian segregation.

The distribution of the transmission rates for *enp1+/−* and *nob1+/−* does not follow any segregation pattern because the male and female gametophyte is affected. For the *enp1* mutant we observed a TR close to zero for selfing, maternal and paternal backcrossing (Table 2); for *nob1+/−* we observed a TR of 1 for selfing and close to zero for maternal transmission of the T-DNA, which is consistent with the observed reduction of seeds per silique and the reduced germination rate. Further, we observed a significantly reduced paternal transmission leading to a TR of 0.34 as well.

**Figure 10:**
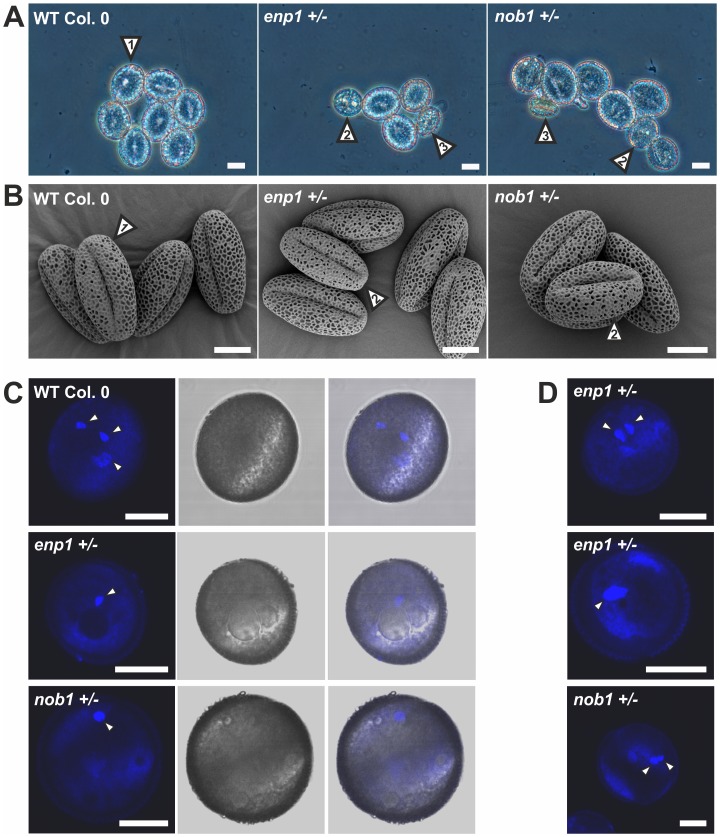
Pollen of *enp1+/−* and *nob1+/−* are delayed in development. A, Pollen of wild type, *enp1+/−* and *nob1+/−* was analyzed with a phase contrast microscope. Scale bars: 10 µM. B, Pollen of wild type and mutant plants were visualized by scanning electron microscopic pictures. Scale bar: 10 µM. C, The pollen was stained with DAPI to visualize the nuclei (left panel). The middle panel shows the DIC image of the pollen, the right panel the overlay of both. White arrows indicate the stained nuclei. Scale bar: 10 µM. D, Additional enp1+/− and nob1+/− pollen is presented to verify the arrest in pollen development. The pollen was stained with DAPI. White arrows indicate the stained nuclei. Scale bar: 10 µM.

Because of the distorted transmission of the T-DNA through the male gametophyte in *nob1+/−* and *enp1+/−*, we analyzed the pollen development in these particular plant lines. We observed pollen delayed in development in both T-DNA insertion lines (Fig. 10A, 2). The delayed pollen is determined by the presence of vacuoles characteristic for the early stages of microgametogenesis shortly after meiosis [64]. The vacuoles are visible as round spheres within the pollen which itself shows a more round morphology in comparison to mature pollen. In the *enp1*+/− mutant the fully developed wild-type pollen (Table 3, Fig. 10A, 1) accounts for 54% of the pollen grains, while 43% are delayed in development (Tab. 3, Fig. 10A, 2) and 3% are crippled with now distinguishable developmental stage (Tab. 3, Fig. 10A, 3). The developmental distortion of the mutant pollen was further investigated by scanning electron microscopy (SEM, Fig. 10B). The pollen from wild-type and *enp1*+/− and *nob1*+/− showed no obvious morphological changes but an equivalent proportion was smaller than wild-type. The round appearance of the delayed pollen is not visible in SEM because the pollen was dried for two day prior to the microscopic analysis.

**Table 3:** Distribution of the distinct pollen shapes representing fully developed (1), delayed (2) and crippled (3) pollen.

mutant line	Shape 1 [%]^1^	Shape 2 [%]	Shape 3 [%]	Total pollen
***Wild type***	100	0	0	300
***enp1.1+*** ***/−***	54±2	43±1	3±1	593
***nob1+*** ***/−***	84±2	14±1	2±1	477

1 Pollen shape according to Figure 9.

To verify the developmental delay in the pollen still containing vacuoles, the nuclei within the pollen grains were stained with DAPI (Fig. 10C,D). Fully developed wild-type pollen contains three nuclei, one vegetative nucleus and two sperm cells nuclei. The pollen in *nob1+/−* and *enp1+/−* still containing vacuoles showed only one or two nuclei and therefore is probably unable to form pollen tubes and carries the T-DNA insertion on its haploid genome.

An exception from the clear full penetration through the female gametophyte is *nob1+/−*. The seed set was reduced by 25% (Tab. 1), about 25% of the seeds appeared pale (Fig. 8) and the TR of selfing plants is reduced due to male and female gametophyte defects (Table 2). Remarkably, about 84% of the pollen of the mutant line has a wild-type shape, while the other mutant-specific shapes occur with a frequency of around 16%. This indicates that sterility of the pollen might lead to the reduced paternal transmission rate of the T-DNA.

## Discussion

### Functional Conservation between Ribosome Biogenesis Factors from Yeast and *Arabidopsis thaliana*

Only limited information on the evolutionary conservation of ribosome biogenesis in plants is available and the information on the few factors investigated in plants give a divergent picture. OsNog2 and ateIf6-1 could complement in the corresponding yeast mutants, while ateIf6-2 or Mtr4 failed to do so [11], [57] Although the factors investigated here show a high sequence similarity (Fig. 1), they did not complement yeast mutants with the exception of atNob1, which somewhat rescued the phenotype (Fig. 1). A reason for the inability of the plant proteins to complement the depletion of their yeast counterpart might be that the plant proteins require different or additional complex partners that are not present in yeast. Another explanation could be that all components of pre-ribosomes are present in yeast and plants, but have differently co-evolved to maintain the functionality of their interaction. However, currently we do not have information about the differences of ribosomal complex composition in different species to verify one of these hypotheses. The observed complementation by Nob1 might be explained by the cytoplasmic action of the protein and by its two domain structure where the largest diversity between the plant and yeast protein (Fig. S1) is restricted to a loop region of unknown function not required for the enzymatic activity [56].

However, the assignment of the plant factors analyzed in here to ribosome biogenesis is supported by several lines of evidences. The intracellular localization of the five factors (Nucleolus: atNoc4, atRrp5, atPwp2; nucleolus & nucleus: atEnp1; nucleus & cytoplasm atNob1; Fig. 3) overlaps with localization of the yeast homologues [40], [42], [59], [65]. Further, atEnp1 and atNob1 co-migrate with pre-ribosomal 40S complexes (Fig. 4), which is comparable to the observed association of scEnp1 with 20S rRNA [65] and the endonuclease activity of atNob1, scNob1 and phNob1 for 20S rRNA (Fig. 4; [35], [37], [56], [59]. In addition, pre-rRNA processing is affected already at the heterozygous state of all lines (Fig. 6). In all investigated heterozygote mutant lines the 35S pre-rRNA was found to be accumulated (Fig. 6), which was also observed while analyzing yeast depletion mutants of *pwp2*, *rrp5* and *noc4* [1], [34], [42]. In the *ENP1* and *NOB1* T-DNA insertion mutants the accumulation of the 35S pre-rRNA is probably due to a general delayed rRNA processing, which cannot be excluded for *pwp2*, *rrp5* and *noc4* as well. The general accumulation of rRNA precursors in plants with defects in ribosome biogenesis is supported by the observation for the Nob1 co-suppression plants. All investigated precursors show an accumulation, but the precursor directly affected by the protein is significantly more enriched (Fig. 7).

Beside obvious similarities between yeast and plant factors, some differences are found. Depletion of Nob1 in yeast leads to an accumulation of pre-rRNAs and a decrease of the mature rRNAs (e.g. [59]). In contrast, in *nob1+/−* the accumulation of the rRNA precursors was rather weak and the level of mature rRNA was not significantly affected, while the P-A3 fragment accumulated (Fig. 6). The latter is surprising because atNob1 shows endonuclease activity and processes the 20S pre-rRNA at site D *in vitro* (Fig. 4). Thus, an accumulation of 20S as observed in yeast was expected. However, the enhanced level of P-A3 and the even reduced level of 20S in *nob1+/−* would indicate that cleavage at site A3 and D is not affected. However, this conclusion is not certain. As seen in the analysis of co-suppression of Nob1, all precursors are enriched to the same extent and only P-A3 shows a higher accumulation. This indicates that in general the 5′ processing of the 18S precursors is affected in the early steps of ribosome biogenesis and influences the downstream processing of the pre-rRNAs accociated with the small ribosomal subunit. Thus, two different explanations for the accumulation of the P-A3 fragment are possible. First, a reduced atNob1 level does not affect the cleavage at site D, but the recruitment of unknown 5′ end processing factors that lead to an accumulation of precursors upstream to 20S pre-rRNA. Second, P-A3 accumulation can occur by disturbed export of pre-ribosomal complexes. It was speculated that in yeast a weak interaction between scNob1 and a putative export adapter for the small subunit scLtv1 exist [66]. A reduction of Nob1 could thereby influence the export competence of the 40S subunit in general. Nevertheless, it is not known, if P-A3 is exported to the cytoplasm. If this is the case it would explain the accumulation of this pre-rRNA in atNob1 deficient plants.

It appears that Nob1 level has to be tightly regulated as the enhanced transcript level in *nob1*+/− triggers a down-regulation of the protein level (Fig. 5). Similarly, the *Arabidopsis* transformation with a wild-type gene under 3S5 promoter leads to a co-suppression of the Nob1 protein (Fig. 7). In this case the 35S driven transcripts are regulated probably by RNAi processing and lead to a reduction of the endogenous and exogenous protein. The requirement for balancing the Nob1 protein level might be explained by “titration” of different factors important for ribosome biogenesis by interaction with Nob1 in the cytoplasm in case of its overexpression.

In summary, a functional of atPwp2, atRrp5, atNoc4, atEnp1 and atNob1 in ribosome biogenesis is supported by the results presented in this study. It was documented that in general they have comparable properties as the yeast factors, but the molecular characteristic appear not to be conserved. Further experiments should aim to the identification of interaction partners of the proteins to finally prove the association of atPwp2, atRrp5, atNoc4, atEnp1 and atNob1 with pre-ribosomal complexes and the functional conservation of the proteins in ribosome biogenesis.

### Mutations in Ribosome Biogenesis Factors Cause Aberrant Gametophyte and Embryo Development

In *Saccharomyces cerevisiae* all five factors in focus of this study are essential. Their homologues in Arabidopsis are essential as well because no homozygous state can be segregated in the T-DNA insertion plant lines (Fig. 5). Consistently with other studies on proteins involved in ribosome biogenesis no growth phenotype was observed for the heterozygote plants (Fig. S6; [8]). Only the silique length was reduced in all T-DNA insertion lines when compared to wild-type (Fig. S8). The reduction in silique length is caused by the abortion of about 50% of the seeds within the siliques of the *PWP2*, *RRP5* and *ENP1* mutants (Table 1, Fig. 8). This phenotype is characteristic for mutations effecting female gametophyte development [67]. For example disruption of *SLOW WALKER1* (*SWA1*), a WD-repeat containing protein involved in the 18S rRNA maturation, leads to a delayed megagametophyte development resulting in asynchronous distribution of ovules in different developmental stages within one pistel [68]. Whether the small undeveloped seeds within the *pwp2+/−*, *rrp5+/−* and *enp1+/−* siliques resemble unfertilized ovules or fertilized ovules arrested early in development has to be addressed in further experiments, but it is tempting to assume that similar effects cause the phenotype observed.

A T-DNA transmission close to zero by female backcrossing was observed for almost all mutants (except *noc4+/−*) indicating an effect of the mutation on female gametophyte development (Table 2). Additionally, a reduction of T-DNA transmission through the male gametophyte was observed in *enp1+/−* and *nob1+/−*. The analysis of pollen properties revealed wild-type like pollen containing three nuclei, but also pollen with two or one nuclei for the two heterozygote lines. This might be explained by a dilution of functional ribosomes from the microspore mother cell leading to an arrest of the microspores before or during the two mitotic cell divisions. During meiosis the microspore mother cell divides into tetrads of haploid microspores whereas two tetrads contain the mutated gene and two the wild type gene. In wild type microspores newly synthesized ribosomes can fulfill their function whereas microspores containing the mutated ribosome biogenesis co-factor gene cannot produce functional ribosomes. Due to cell growth and cell division the pollen grains have a high demand of ribosomes which cannot be covered by the ribosomes inherited from the microspore mother cell. Consequently, the development of the pollen grain is delayed and eventually aborted.

A similar argumentation can be build up for the globally observed defect of reduction of T-DNA transmission through the male gametophyte. Although an arrest or delay in the mitotic cell divisions of the female gametophyte was not proven earlier studies reported a disturbed development of the megaspore during mitosis [14]. Assuming that the aborted ovules in the siliques of the T-DNA insertion lines analyzed here are also defective in mitotic cell cycle progression, the arrest of the female gametophyte in the mitotic cell divisions could be due to ribosome dilution during gametogenesis as well. The larger cell size, the increased number of mitotic cell divisions and/or the cellularization processes that occur in ovules shortly before fertilization might account for a higher demand on ribosome biogenesis related genes on the development of the female gametophyte in comparison to the male gametophyte.

In summary, the results show the great importance of functional ribosome biogenesis on plant development. The deletion of co-factors involved in ribosome biogenesis disturbs cell cycle progression and cell proliferation especially in the haploid stages of plant development. The mutations in ribosome biogenesis co-factors mainly affect the mitotic cell cycle progression, which is supported by the distribution of the T-DNA transmission through the male and female gametophyte. A typical feature of mitotic mutants is the non-Mendelian segregation pattern. In contrast meiotic mutants are sporophytic mutations and would show normal Mendelian segregation patterns at a heterozygous state [67]. Most interestingly, the deletion of different ribosome biogenesis co-factors, although these proteins participate in the same cellular process, led to diverse defects in gametophyte or embryo development represented by various segregation patterns. Whereas a disruption of at*RRP5* and at*PWP2* lead to exclusively female gametophyte defects, at*ENP1* and at*NOB1* mutants show female and male gametophyte defects. A clear exception is the T-DNA insertion line of at*NOC4* where a normal Mendelian segregation occurs but the homozygous embryos arrest in the globular stage of development. These diverse defects of proteins participating in the same pathway are surprising and might be explainable by epigenetic modifications (Fig. S9; [69]).

## Supporting Information

Figure S1 **Alignment between yeast and plant protein sequences.**
(DOCX)

Figure S2 **Confirmation of expression of atEnp1 and atNob1 in yeast depletion strains.**
(DOCX)

Figure S3 **Transcript abundance of the genes of interests and the reference gene** ***UBI3*** **without normalization.**
(DOCX)

Figure S4 **Expression analysis with genevestigator.**
(DOCX)

Figure S5 **Confirmation of the specificity of the antibodies against atEnp1 and atNob1.**
(DOCX)

Figure S6 **Growth analysis of wild-type and heterozygous lines.**
(DOCX)

Figure S7 **Quantification of the P-A3, P1-A3 and 20S transcript in different genotypes.**
(DOCX)

Figure S8 **Size distribution of siliques from wild-type and heterozygote lines.**
(DOCX)

Figure S9 **Properties of the genomic DNA.**
(DOCX)

Table S1 **Border sequences of T-DNA insertions.**
(DOCX)

Table S2 **Oligonucleotides used in this study.**
(DOCX)

Table S3 **Probes for northern blot analysis.**
(DOCX)

Table S4 **Statistical evaluation of transmission rates.**
(DOCX)
