# Supplementary material for: 40S Ribosome Biogenesis Co-Factors Are Essential for Gametophyte and Embryo Development
Source: PLoS One. 2013 Jan 30;8(1):e54084. doi: 10.1371/journal.pone.0054084 (PMC3559688; doi:10.1371/journal.pone.0054084)
Supplement: Figure S1 — Alignment between yeast and plant protein sequences. (DOCX) [file pone.0054084.s001.docx]

**Figure S1.** Alignment between yeast and plant protein sequences.

A, Rrp5. B, Pwp2. C, Noc4. D, Nob1. E, Enp1.

**A**

AT3G11964 MVVPQKKFANGKRNDSTKSFKPMKKPFKKTKDDVAARSEAMALQLEDVPDFPRGGGTSLS

RRP5 MVA------------STKRKRDEDFPLSREDSTKQPSTSSLVRNTEEV-SFPRGGASALT

**. *** : . *:.: .. . :.::. : *:* .*****.::*:

AT3G11964 KKEREKLYEEV-------DAEFDADERVSKKSKGGKSKKRIPSDLDDLGLLFGGGLHGKR

RRP5 PLELKQVANEAASDVLFGNESVKASEPASRPLKKKKTTKKSTSKDSEASSANSDEARAGL

* ::: :*. : ...*.* .*: * *:.*: .*. .: . .. :.

AT3G11964 PRYANKITTKNISPGMKLLGVVTEVNQKDIVISLPGGLRGLVRASEVSDFTDRGIEDDEN

RRP5 IEHVNFKTLKN---GSSLLGQISAITKQDLCITFTDGISGYVNLTHISEEFTSILEDLDE

.:.* * ** * .*** :: :.::*: *::..*: * *. :.:*: :** ::

AT3G11964 ELLGDI-------------------------------------FSVGQLVPCIVLE---L

RRP5 DMDSDTDAADEKKSKVEDAEYESSDDEDEKLDKSNELPNLRRYFHIGQWLRCSVIKNTSL

:: .* * :** : * *:: *

AT3G11964 DDDKKEAGKRKIWLSLRLSLLHKGFSFDSFQLGMVFSANVKSIEDHGSILHFGLPSITGF

RRP5 EPSTKKSKKKRIELTIEPSSVNI-YADEDLVKSTSIQCAVKSIEDHGATLDVGLPGFTGF

: ..*:: *::* *::. * :: :: :.: . :.. ********: *..***.:***

AT3G11964 IEISDDGNQESGMKTGQLIQGVVTKIDRDRKIVHLSSDPDSVAKCLTKDLSGMSFDLLIP

RRP5 IAKKDFGNFEK-LLPGAVFLGNITK-KSDRSIVVNTDFSDKKNKITQIS----SIDAIIP

* .* ** *. : .* :: * :** . **.** :. .*. * . *:* :**

AT3G11964 GMMVNARVQSVLENGILFDFLTYFNGTVDLFHLKNPLSNKSWKDEYNQNKTVNARILFI-

RRP5 GQIVDLLCESITKNGIAGKVFGLVSGVVNVSHLRT-FSEEDLKHKFVIGSSIRCRIIACL

* :*: :*: :*** ..: ..*.*:: **:. :*::. *.:: ..::..**:

AT3G11964 --DPSSRAVGLTLSPHVV-----CNKAPPLHVFS-GDIFDEAKVVRIDKSGLLLELPSK-

RRP5 ENKSGDKVLILSNLPHILKLEDALRSTEGLDAFPIGYTFESCSIKGRDSEYLYLALDDDR

....:.: *: **:: ..: *..*. * *:...: *.. * * * ..

AT3G11964 --PTPTPAYVSFKEGNHIRVRVLGLKQMEGLAVGTLKESAFEGPVFTHSDVKPG-MVTKA

RRP5 LGKVHSSRVGEIENSENLSSRVLGYSPVDDIYQLSTDPKYLKLKYLRTNDIPIGELLPSC

. :. .:::.::: **** . ::.: : . . :: : .*: * ::...

AT3G11964 KVISVDTFGAIVQ-FSGGLKAMCPLRHMSEFEVTKPRKKFKVGAELVFRVLGCKSK-RIT

RRP5 EITSVSSSGIELKIFNGQFKASVPPLHISDTRLVYPERKFKIGSKVKGRVISVNSRGNVH

:: **.: * :: *.* :** * *:*: .:. *.:***:*::: **:. :*: .:

AT3G11964 VTYKKTLVK---SKLPILSSYTDA----TEGLVTHGWITKIEKHGCFVRFYNGVQGFVPR

RRP5 VTLKKSLVNIEDNELPLVSTYENAKNIKEKNEKTLATIQVFKPNGCIISFFGGLSGFLPN

** **:**: .:**::*:* :* :. * . * :: :**:: *:.*:.**:*.

AT3G11964 FELGLEPGSDPDSVFHVGEVV----------------KCRVTSAVHGTQRITLNDSIKLG

RRP5 SEISEVFVKRPEEHLRLGQTVIVKLLDVDADRRRIIATCKVSNEQAAQQKDTIENIVPGR

*:. . *:. :::*:.* .*:*:. . *: *::: :

AT3G11964 SIVSGIIDTITSQAVIVRVKSKSVVKGTISAEHLADHH-EQAKLIMSLLRPGYELDKLLV

RRP5 TIITVHVIEKTKDSVIVEIPDVG-LRGVIYVGHLSDSRIEQNRAQLKKLRIGTELTGLVI

:*:: : *.::***.: . . ::*.* . **:* : ** : :. ** * ** *::

AT3G11964 -LDIEGNNMALSSKYSLIKLA--EELP---SDFNQLQPNSVVHGYVCNLIENGCFVRFLG

RRP5 DKDTRTRVFNMSLKSSLIKDAKKETLPLTYDDVKDLNKDVPMHAYIKSISDKGLFVAFNG

* . . : :* * **** * * ** .*.::*: : :*.*: .: ::* ** * *

AT3G11964 RLTGFAPRSKAIDDPKADVSESFFVGQSVRANIVDVNQEKSRITLSLKQSSCASVDASFV

RRP5 KFIGLVLPSYAVDSRDIDISKAFYINQSVTVYLLRTDDKNQKFLLSLKAPKV--------

:: *:. * *:*. . *:*::*::.*** . :: .::::.:: **** ..

AT3G11964 QEYFLMDEKISDLQSSDITKSDCSWVEKFSIGSLIKGTIQEQNDLGVVVNFDNINNVLGF

RRP5 ------------------------------------------------------------

AT3G11964 IPQHHMGGATLVPGSVVNAVVLDISRAERLVDLSLRPELLNNLTKEVSNSSKKKRKRGIS

RRP5 ----------------------------------------------------KEEKKKVE

*:.*: :.

AT3G11964 KELEVHQRVSAVVEIVKEQHLVLSIPEHGYTIGYASVSDYNTQKLPVKQFSTGQSVVASV

RRP5 SNIE-------------------------------------------------DPVDSSI

.::* :.* :*:

AT3G11964 KAVQNPLTSGRLLLLLDSVSGTSETSRSKRAKKKSSCEVGSVVHAEITEIKPFELRVNFG

RRP5 KSWDD-------------------------------LSIGSIVKAKIKSVKKNQLNVILA

*: :: .:**:*:*:*..:* :*.* :.

AT3G11964 NSFRGRIHITEVLVNDASTSD--EPFAKFRVGQSISARVVAK---------PCHTDIKKT

RRP5 ANLHGRVDIAEVFDTYEEITDKKQPLSNYKKDDVIKVKIIGNHDVKSHKFLPITHKISKA

.::**:.*:**: . . :* :*::::: .: *..:::.: * .*.*:

AT3G11964 QLWELSVKPAMLKDSSEFNDTQESEQLEFAAGQCVIGYVYKVDKEWVWLAVSRNVTARIF

RRP5 SVLELSMKPSELKSKEVHTKSLE----EINIGQELTGFVNNSSGNHLWLTISPVLKARIS

.: ***:**: **... ...: * *: ** : *:* : . : :**::* :.***

AT3G11964 ILDTSCKAHEL-EEFERRFPIGKAVSGYVLTYNKEKKTLRLVQRPLLFIHKSIANGGGSK

RRP5 LLDLADNDSNFSENIESVFPLGSALQVKVASIDREHGFVNAIGKSHVDINMST-------

:** : : :: *::* **:*.*:. * : ::*: :. : :. : *: *

AT3G11964 TDKPDSSIPGDDDTLFIHEGDILGGRISKILPGVGGLRVQLGPYVFGRVHFTEINDSWVP

RRP5 ----------------IKVGDELPGRVLKIAEKY--VLLDLGNKVTGISFITDALNDFSL

*: ** * **: ** : ::** * * .:*: :.:

AT3G11964 DPLDGFRE--GQFVKCKVLEISSSSKGTWQIELSLRTSLDGMSSADHLSEDLKNNDNVCK

RRP5 TLKEAFEDKINNVIPTTVLSVDEQNK---KIELSLRPATAKTRS----------------

:.*.: .:.: .**.:....* :******.: *

AT3G11964 RFERIEDLSPDMGVQGYVKNTMSKGCFIILSRTVEAKVRLSNLCDTFVKEPEKEFPVGKL

RRP5 -IKSHEDLKQGEIVDGIVKNVNDKGIFVYLSRKVEAFVPVSKLSDSYLKEWKKFYKPMQY

:: ***. . *:* ***. .** *: ***.*** * :*:*.*:::** :* : :

AT3G11964 VTGRVLNVEPLSKRIEVTLKTVNAGGRPKSESYDLKKL------HVGDMISGRIRRVEPF

RRP5 VLGKVVTCDEDS-RISLTLRESEING-------DLKVLKTYSDIKAGDVFEGTIKSVTDF

* *:*:. : * **.:**: : .* *** * :.**::.* *: * *

AT3G11964 GLFIDIDQT-GMVGLCHISQLSDDRMENVQARYKAGESVRAKILKLDEEKKRISLGMKSS

RRP5 GVFVKLDNTVNVTGLAHITEIADKKPEDLSALFGVGDRVKAIVLKTNPEKKQISLSLKAS

*:*:.:*:* .:.**.**::::*.: *::.* : .*: *:* :** : ***:***.:*:*

AT3G11964 YLMNGDDDKAQPLSEDNTSMECDPI-NDPKSEVLAAVDDFGFQETSGGTSLVLAQVESRA

RRP5 HF----SKEAELASTTTTTTTVDQLEKEDEDEVMA---DAGFNDSDSESDIGDQNTEVAD

:: ..:*: * .*: * : :: :.**:* * **:::.. :.: :.*

AT3G11964 SIPPLEVD--------------LDDIEETDFDSSQNQEKLLGANKDEKSKRREKQKDKEE

RRP5 RKPETSSDGLSLSAGFDWTASILDQAQEEE-ESDQDQED-FTENKKHKHKRR---KENVV

* . * **: :* : :*.*:**. : **..* *** *::

AT3G11964 REKKIQAAEGRLLEHHAPENADEFEKLVRSSPNSSFVWIKYMAFMLSLADIEKARSIAER

RRP5 QDKTID------INTRAPESVADFERLLIGNPNSSVVWMNYMAFQLQLSEIEKARELAER

::*.*: :: :***.. :**:*: ..****.**::**** *.*::*****.:***

AT3G11964 ALRTINIREEEEKLNIWVAYFNLENEHGNPPEESVKKVFERARQYCDPKKVYLALLGVYE

RRP5 ALKTINFREEAEKLNIWIAMLNLENTFGT--EETLEEVFSRACQYMDSYTIHTKLLGIYE

**:***:*** ******:* :**** .*. **::::**.** ** *. .:: ***:**

AT3G11964 RTEQYKLADKLLDEMIKKF-KQSCKIWLRKIQSSLKQNEE-AIQSVVNRALLCLPRHKHI

RRP5 ISEKFDKAAELFKATAKKFGGEKVSIWVSWGDFLISHNEEQEARTILGNALKALPKRNHI

:*::. * :*:. *** :. .**: : :.:*** ::::..** .**:::**

AT3G11964 KFISQTAILEFKCGVADRGRSLFEGVLREYPKRTDLWSVYLDQEIRLGEDDVIRSLFERA

RRP5 EVVRKFAQLEFAKGDPERGRSLFEGLVADAPKRIDLWNVYVDQEVKAKDKKKVEDLFERI

:.: : * *** * .:********:: : *** ***.**:***:: :.. :..****

AT3G11964 ISLSLPPKKMKFLFKKFLEYEKSVGDEERVEYVKQRAMEYANSTLA-----

RRP5 ITKKITRKQAKFFFNKWLQFEESEGDEKTIEYVKAKATEYVASHESQKADE

*: .:. *: **:*:*:*::*:* ***: :**** :* **. * :

**B**

AT1G15440 M--EFRFENLLGAPYRGGNAVITKN-TQLISPVGNRVSVTDLSKNHSVTLPLETSTNICR

PWP2 MKSDFKFSNLLGTVYRQGNITFSDDGKQLLSPVGNRVSVFDLINNKSFTFEYEHRKNIAA

* :*:*.****: ** ** .::.: .**:********* ** :*:*.*: * .**.

AT1G15440 LASSPDGTFLLAVDEQNRCLFINLPRRVVLHRITFKDKVGALKFSPNGKFIAVGIGKLVE

PWP2 IDLNKQGTLLISIDEDGRAILVNFKARNVLHHFNFKEKCSAVKFSPDGRLFALASGRFLQ

: . :**:*:::**:.*.:::*: * ***::.**:* .*:****:*:::*:. *::::

AT1G15440 IWRSPGFR--RAVLPFERVRTFANSDDKVVSLEWSLDSDYLLVGSRDLAARLFCVRKLKG

PWP2 IWKTPDVNKDRQFAPFVRHRVHAGHFQDITSLTWSQDSRFILTTSKDLSAKIWSVDSEEK

**::*... * . ** * *..*. :.:.** ** ** ::*. *:**:*:::.* . :

AT1G15440 VLNKPFLFLGHRDSVVGCFFGVDKMTNKVNRAFTIARDGYIFSWGYTEKDVKMDESEDGH

PWP2 NL-AATTFNGHRDYVMGAFFSHDQ-----EKIYTVSKDGAVFVWEFTKRPSDDDDNE---

* . * **** *:*.**. *: :: :*:::** :* * :*:: . *:.*

AT1G15440 SEPPSPVTPDRADEVMVENGGGVGTELKKRKEYDGKGLESDEEGDDDDEEYMHRGKWVLL

PWP2 ----------------------------------------SEDDDKQEEVDISKYSWRIT

.*:.*.::* : : .* :

AT1G15440 RKDGCNQASAKVTACDYHQGLDMVVVGFSNGVFGLYQMPDFICIHLLSISRQKLTTAVFN

PWP2 KKHFFYANQAKVKCVTFHPATRLLAVGFTSGEFRLYDLPDFTLIQQLSMGQNPVNTVSVN

:*. .***.. :* . ::.***:.* * **::*** *: **:.:: :.*. .*

AT1G15440 ERGNWLTFGCAKLGQLLVWDWRTETYILKQQGHYFDVNCVTYSPDSQLLATGADDNKVKV

PWP2 QTGEWLAFGSSKLGQLLVYEWQSESYILKQQGHFDSTNSLAYSPDGSRVVTASEDGKIKV

: *:**:**.:*******::*::*:********: ..*.::****.. :.*.::*.*:**

AT1G15440 WNVMSGTCFITFTEHTNAVTALHFMADNHSLLSASLDGTVRAWDFKRYKNYKTYTTPTPR

PWP2 WDITSGFCLATFEEHTSSVTAVQFAKRGQVMFSSSLDGTVRAWDLIRYRNFRTFTGTERI

*:: ** *: ** ***.:***::* .: ::*:**********: **:*::*:* .

AT1G15440 QFVSLTADPSGDVVCAGTLDSFEIFVWSKKTGQIKDILSGHEAPVHGLMFSPLTQLLASS

PWP2 QFNCLAVDPSGEVVCAGSLDNFDIHVWSVQTGQLLDALSGHEGPVSCLSFSQENSVLASA

** .*:.****:*****:**.*:*.*** :***: * *****.** * ** ..:***:

AT1G15440 SWDYTVRLWDVFASKGTVETFRHNHDVLTVAFRPDGKQLASSTLDGQINFWDTIEGVLMY

PWP2 SWDKTIRIWSIFGRSQQVEPIEVYSDVLALSMRPDGKEVAVSTLKGQISIFNIEDAKQVG

*** *:*:*.:*. . **.:. ***::::*****::* ***.***.::: :. :

AT1G15440 TIEGRRDIAGGRVMTDRRSAANSSSGKCFTTLCYSADGGYILAAGTSRYICMYDIADQVL

PWP2 NIDCRKDIISGRFNQDRFTAKNSERSKFFTTIHYSFDGMAIVAGGNNNSICLYDVPNEVL

.*: *:** .**. ** :* **. .* ***: ** ** *:*.*... **:**:.::**

AT1G15440 LRRFQISHNLSLDGVLDFLHSKKMTEAGPIDLIDD--DNSDEEGGIDKQSRGNLGYDLPG

PWP2 LKRFIVSRNMALNGTLEFLNSKKMTEAGSLDLIDDAGENSDLEDRIDN--------SLPG

*:** :*:*::*:*.*:**:********.:***** :*** *. **: .***

AT1G15440 SR------PNRGRPIIRTKSLSIAPTGRSFAAATTEGVLIFSIDDTFIFDPTDLDIDVTP

PWP2 SQRGGDLSTRKMRPEVRVTSVQFSPTANAFAAASTEGLLIYSTNDTILFDPFDLDVDVTP

*: ..: ** :*..*:.::**..:****:***:**:* :**::*** ***:****

AT1G15440 EAVEAAIEEDEVSRALALSMRLNEDSLIKKCIFAVAPADIKAVAISVRQKYLERLMEALV

PWP2 HSTVEALREKQFLNALVMAFRLNEEYLINKVYEAIPIKEIPLVASNIPAIYLPRILKFIG

.:. *:.*.:. .**.:::****: **:* *:. :* ** .: ** *::: :

AT1G15440 DLLENCPHLEFILHWCQEICKAHGSSIQRNYRTLLPALRSLQKAITRAHQDLADMCSSNE

PWP2 DFAIESQHIEFNLIWIKALLSASGGYINEHKYLFSTAMRSIQRFIVRVAKEVVNTTTDNK

*: :. *:** * * : : .* *. *:.: : .*:**:*: *.*. :::.: :.*:

AT1G15440 YTLRYLCSVPNNH-----------------------------------------------

PWP2 YTYRFLVSTDGSMEDGAADDDEVLLKDDADEDNEENEENDVVMESDDEEGWIGFNGKDNK

** *:* *. ..

AT1G15440 --------------------

PWP2 LPLSNENDSSDEEENEKELP

**C**

AT2G17250 MASILSKKQKKNEKYTLKELKSLGHDLLTS--RSHINN-LPLLLTFVSPESPPQFVVES-

Noc4 MVLL------------ISEIKDIAKRLTAAGDRKQYNSIIKLINELVIPENVTQLEEDET

*. : :.*:*.:.: * :: *.: *. : *: :* **. .*: :.

AT2G17250 -------LLSLQSFFTPLLSQLPPTSSSPSSTKTEDPEVVFKAWLRSKFDEFVKLLLDVL

Noc4 EKNLRFLVMSLFQIFRKLFSRGDLT--LPSSKKSTLEKEQFVNWCRKVYEAFKTKLLAII

::** .:* *:*: * ***.*: : * * *. :: * . ** ::

AT2G17250 VSQQSEDSLRGIVLGTLMEFVKLLN--------AGRFHSSIYHRLLDAIIHSEV-DIE--

Noc4 SDIPFETSLGLDSLDVYLQLAELESTHFASEKGAPFFPNKTFRKLIIALWSSNMGEIEDV

. * ** *.. :::.:* . * * .. :::*: *: *:: :**

AT2G17250 ---------IFLDILTSKYFKYIDVRYFTYISMEKFVK------TLEASVSADRTVIENN

Noc4 KSSGASENLIIVEFTEKYYTKFADIQYYFQSEFNQLLEDPAYQDLLLKNVGKWLALVNHD

*:::: . * *: *::*: .:::::: * .*. ::::::

AT2G17250 EAESDSKESLELSVRKIYQVLSQIPPPEKQAEKSQHEMWSGSDESISEKPTDKKKKTEKG

Noc4 KHCSSVDADLEIFV----------PNPPQAIEN---------------------------

: *. . .**: * * * : *:

AT2G17250 DSTLLSPATISKRMKLKFTKAWISFLRLPLPIDVYKEVLASIHLTVIPHLSNPTMLCDFL

Noc4 ----------ESKFKSNFEKNWLSLLNGQLSLQQYKSILLILHKRIIPHFHTPTKLMDFL

..::* :* * *:*:*. *.:: **.:* :* :***: .** * ***

AT2G17250 TKSYDI------GGVVSVMALSSLFILMTQHGLEYPFFYEKLYALLVPSVFVAKHRAKFL

Noc4 TDSYNLQSSNKNAGVVPILALNGLFELMKRFNLEYPNFYMKLYQIINPDLMHVKYRARFF

*.**:: .***.::**..** **.:..**** ** *** :: *.:: .*:**:*:

AT2G17250 QLLDACLKSSMLPAYLAASFTKKLSRLSLSIPPAGSLVITALIYNLLRRNPTINHLVQEI

Noc4 RLMDVFLSSTHLSAHLVASFIKKLARLTLESPPSAIVTVIPFIYNLIRKHPNCMIMLH--

:*:*. *.*: *.*:*.*** ***:**:*. **:. :.: .:****:*::*. :::

AT2G17250 VENADEANTEAGEHNESQPKTIKKRKLGIDYFNNQESDPKKSGALKSSLWEIDTLRHHYC

Noc4 -NPAFISNPFQTPDQVANLKTLKENY--VDPFDVHESDPELTHALDSSLWELASLMEHYH

: * :*. .: :: **:*:. :* *: :****: : **.*****: :* .**

AT2G17250 PPVSRFISSLETNLTIRSKTTEMKIEDFCSGSYATIFGDEIRRRVKQVPLAFYKTVPTSL

Noc4 PNV----ATLAKIFAQPFKKLSYNMEDFLDWNYDSLLNAESSRKLKTLPTLEFEAF-TNV

* * ::* . :: *. . ::*** . .* :::. * *::* :* :::. *.:

AT2G17250 FADSD------------FPG--WTFTIPQEEGTC

Noc4 FDNEDGDSEASSQGNVYLPGVAW-----------

* :.* :** *

**D**

AT5G41190 MDPKPTSMWSSIVKKDPPSKPPVNDGAPAAILGMVGNCKSTKGISIAVVDANAII-EGRQ

Nob1 MTE-----------------------------------NQTAHVRALILDATPLITQSYT

* :.* : ::**..:* :.

AT5G41190 SLTNFADKFVTVPEVLSEIRDPASRRRLAFIPF--TIDTMEPSPESLSKVIKFARATGDL

Nob1 HYQNYAQSFYTTPTVFQEIKDAQARKNLEIWQSLGTLKLVHPSENSIAKVSTFAKLTGDY

*:*:.* *.* *:.**:*. :*:.* : *:. :.** :*::** .**: ***

AT5G41190 QSLSDVDLKLIALSYTLEAQVYGTKNLRDVPPPIQTVRVKRLPEKDLPGWGSNVANLEEW

Nob1 SVLSANDLHILALTYELEIKLNNGD-----------WRLRKKPGDALDASKADVGTDGKQ

. ** **:::**:* ** :: . . *::: * . * . ::*.. :

AT5G41190 EALE-NETEEKSNANSKILPLKDLNMNIIASDNVSEVGSVVSHTENHEEDVQEGGKKHRR

Nob1 KLTEDNKKEEDSESVPK--------------------------------------KKNKR

: * *:.**.*:: .* **::*

AT5G41190 YPPKKTEIKLEGKMVVEGVDASQGQYDDDDDASDWRPAVSRSTHSKYLRRKARWEHYNAL

Nob1 RGGKKQKAKREAREAREAENANL-----------------------ELESKAEEHVEEAG

** : * *.: . *. :*. *. **. . :*

AT5G41190 AEQEIQKDQEADKARHTKEANETHAKDSGKNGEDISSILKDMRLEEESLRALQEETEETN

Nob1 SKEQICNDENI------------------KESSDLNEVFED-------------------

::::* :*:: *:..*:..:::*

AT5G41190 AEATLINGEDDIDHDIEVEAEGIDVANQALENLEIASEAEDTFEASSIGDDGSSEQSWSL

Nob1 -------ADDDGDW----------ITPENLTEAIIKDSGEDT--TGSLGVEASEEDRHVA

.:** * :: : * : * ...*** :.*:* :.*.*:

AT5G41190 RALSESSVACITGDYAMQNVILQMGLRLLA-PGGMQIRQLHRWILKCHACYTVTP----E

Nob1 LNRPENQVALATGDFAVQNVALQMNLNLMNFMSGLKIKRIRNYMLRCHACFKIFPLPKDG

.*..** ***:*:*** ***.*.*: .*::*::::.::*:****:.: *

AT5G41190 IGRIFCPKCGNGGTLRKVAVTIGA-NGAIIAACKPRITL--RGTQYSI--PMPKGGREAI

Nob1 KPKHFCASCGGQGTLLRCAVSVDSRTGNVTPHLKSNFQWNNRGNRYSVASPLSKNSQKRY

: **..**. *** : **::.: .* : . *..: **.:**: *:.*..::

AT5G41190 TK----------NLILREDQ------LPQKLLHPRTKKK------ASKPGDEYFVSDDVF

Nob1 GKKGHVHSKPQENVILREDQKEYEKVIKQEEWTRRHNEKILNNWIGGGSADNYISPFAIT

* *:****** : *: * ::* .. ..*:*: . :

AT5G41190 -LNHHSDRKAPLQPPVRKAMSVFSQKRNPNDNHYSRSMH

Nob1 GLKQHNVR-------IGKGRYVNSSKR--------RS--

*::*. * : *. * *.** **

**E**

AT1G31660 M---AKKRDRIVNTQPFISDDASVASSRKRSKVPKTHQ----------KQEKLIEAGMSE

ENP1 MARASSTKARKQRHDPLLKDLDAAQGTLKKINKKKLAQNDAANHDAANEEDGYIDSKASR

* :..: * . :*::.* :. .: *: : * * ::: *:: *.

AT1G31660 KIMKQALAQQKEVADEENAERNPSSAAFAVAGAATAGEEQKILEEEEDDIDDFDGTFENQ

ENP1 KILQLAKEQQDEIEGEELAESERNKQFEARFTTMSYDDEDEDEDEDEEAFGEDISDFEPE

**:: * **.*: .** ** : .. * : : .:*:: :*:*: :.: . ** :

AT1G31660 SQFDKQE---EINEDDEKLFESFLNK-----NAPPQRTLTDIIIKKLKDKDADLAEEERP

ENP1 GDYKEEEEIVEIDEEDAAMFEQYFKKSDDFNSLSGSYNLADKIMASIREKESQVEDMQDD

.::.::* **:*:* :**.:::* . . . .*:* *: .:::*:::: : :

AT1G31660 DP----------------------KMDPAITKLYKGVGKFMSEYTVGKLPKAFKLVTSME

ENP1 EPLANEQNTSRGNISSGLKSGEGVALPEKVIKAYTTVGSILKTWTHGKLPKLFKVIPSLR

:* : : * *. **.::. :* ***** **::.*:.

AT1G31660 HWEDVLYLTEPEKWSPNALYQATRIFASNLKDRQVQRFYNYVLLPRVREDIRKHK--KLH

ENP1 NWQDVIYVTNPEEWSPHVVYEATKLFVSNLTAKESQKFINLILLERFRDNIETSEDHSLN

:*:**:*:*:**:***:.:*:**::*.***. :: *:* * :** *.*::*.. : .*:

AT1G31660 FALYQALKKSLYKPSAFNQGILFPLCKSGTCNLREAVIIGSILEKCSIPMLHSCVALNRL

ENP1 YHIYRAVKKSLYKPSAFFKGFLFPLVETG-CNVREATIAGSVLAKVSVPALHSSAALSYL

: :*:*:********** :*:**** ::* **:***.* **:* * *:* ***..**. *

AT1G31660 AEMDYCGTTSYFIKVLLEKKYCMPYRVLDALVAHFMRF---------VDDIRVMPVIWHQ

ENP1 LRLPFSPPTTVFIKILLDKKYALPYQTVDDCVYYFMRFRILDDGSNGEDATRVLPVIWHK

.: :. .*: ***:**:***.:**:.:* * :**** * **:*****:

AT1G31660 SLLTFVQRYKYEILKEDKEHLQTLLQRQKHHLVTPEILRELKDSRNR------GEKEDPM

ENP1 AFLTFAQRYKNDITQDQRDFLLETVRQRGHKDIGPEIRRELLAGASREFVDPQEANDDLM

::***.**** :* :::::.* :::: *: : *** *** . .* ::* *

AT1G31660 VDNFAPVPAKEDRFDIPEVPMEED

ENP1 ID--------------------VN

:* :

**Fig S1**
