# Supplementary material for: 40S Ribosome Biogenesis Co-Factors Are Essential for Gametophyte and Embryo Development
Source: PLoS One. 2013 Jan 30;8(1):e54084. doi: 10.1371/journal.pone.0054084 (PMC3559688; doi:10.1371/journal.pone.0054084)

**Figure S2.** Confirmation of expression of atEnp1 and atNob1 in yeast depletion strains.

The expression of atNob1 (upper panel) and atEnp1 (lower panel) in the corresponding yeast depletion strains was determined by western blot analysis. As a control Arabidopsis cell culture extract was used. The equal protein amount loaded on the gel was confirmed by Ponceau staining.


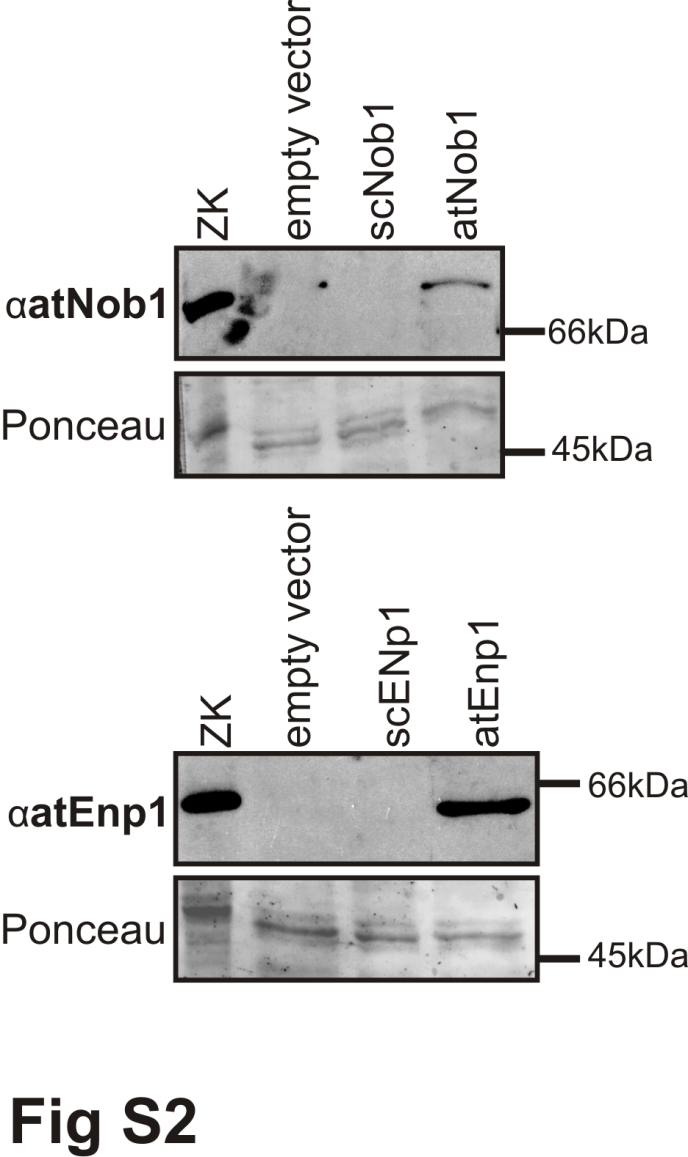

Supplement: Figure S2 — Confirmation of expression of atEnp1 and atNob1 in yeast depletion strains. (DOCX) [file pone.0054084.s002.docx]
