# Supplementary material for: 40S Ribosome Biogenesis Co-Factors Are Essential for Gametophyte and Embryo Development
Source: PLoS One. 2013 Jan 30;8(1):e54084. doi: 10.1371/journal.pone.0054084 (PMC3559688; doi:10.1371/journal.pone.0054084)

**Figure S3.** Transcript abundance of the genes of interests and the reference gene *UBI3* without normalization.

Transcript abundance of the five selected genes and the reference gene *UBI3* was determined by quantitative RT-PCR. Equal amounts of RNA were added to the RT reaction. Three independent experiments were used to determine the standard deviations.


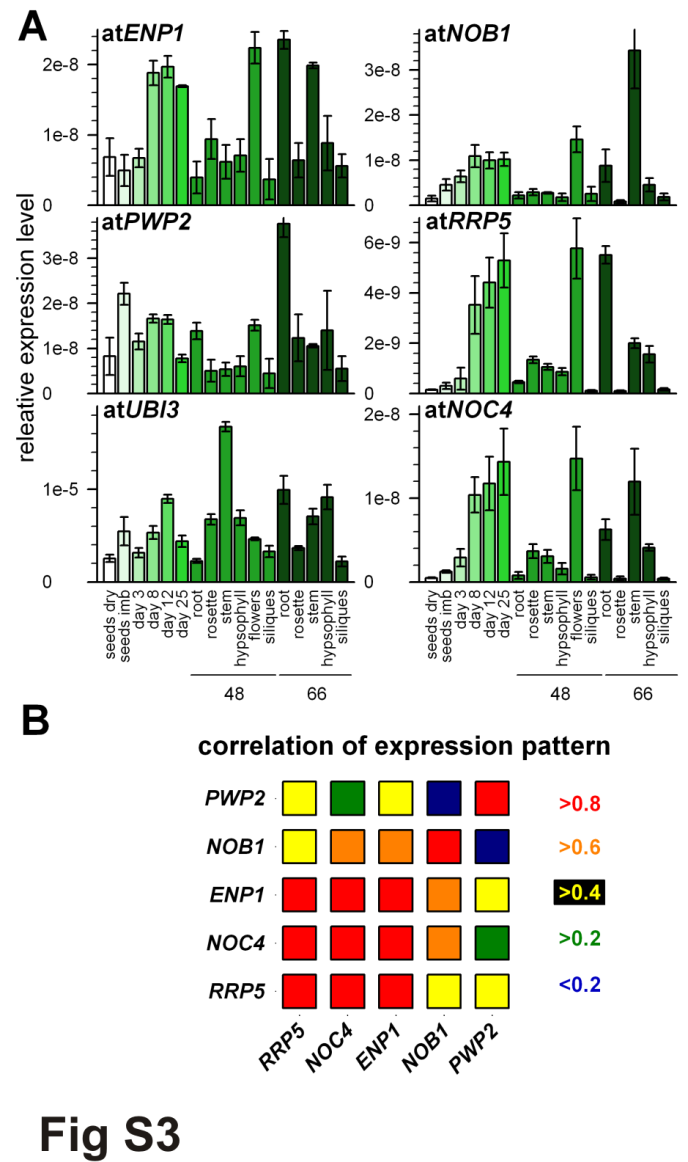

Supplement: Figure S3 — Transcript abundance of the genes of interests and the reference gene UBI3 without normalization. (DOCX) [file pone.0054084.s003.docx]
