# Supplementary material for: 40S Ribosome Biogenesis Co-Factors Are Essential for Gametophyte and Embryo Development
Source: PLoS One. 2013 Jan 30;8(1):e54084. doi: 10.1371/journal.pone.0054084 (PMC3559688; doi:10.1371/journal.pone.0054084)

**Figure S4.** Expression analysis with genevestigator.

The five genes were submitted to the genevestigator search engine (https://www.genevestigator.com/gv/plant.jsp) to determine the expression patterns in different Arabidopsis tissues. The ATH1: 22k array was used and the different expression levels were visualized by a heat map.


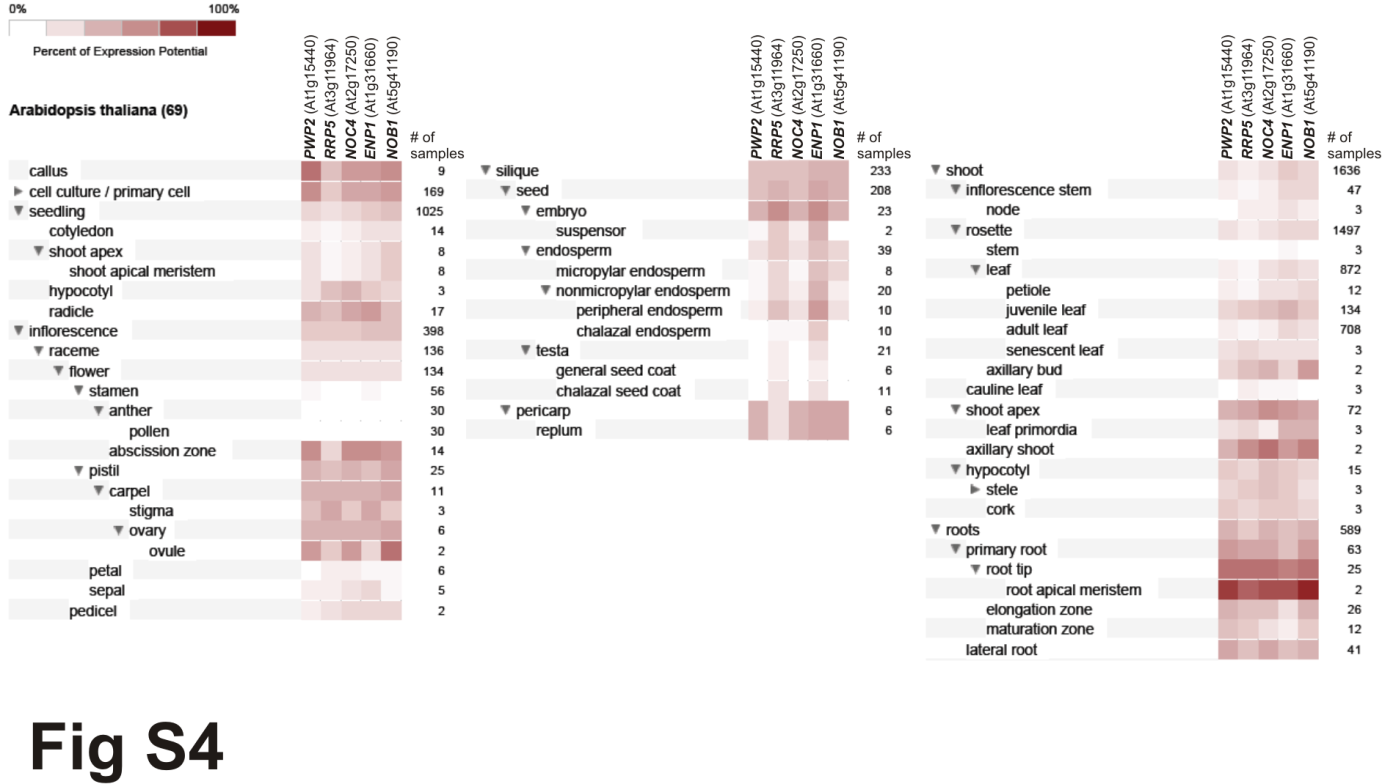

Supplement: Figure S4 — Expression analysis with genevestigator. (DOCX) [file pone.0054084.s004.docx]
