# Supplementary material for: 40S Ribosome Biogenesis Co-Factors Are Essential for Gametophyte and Embryo Development
Source: PLoS One. 2013 Jan 30;8(1):e54084. doi: 10.1371/journal.pone.0054084 (PMC3559688; doi:10.1371/journal.pone.0054084)

**Figure S5.** Confirmation of the specificity of the antibodies against atEnp1 and atNob1.

Antibodies against the corresponding proteins were tested by immunodecoration of recombinant protein (lane 1), cell culture extract (lane 2) and flower extract (lane 3). The asterisks indicate degradation products of atNob1.


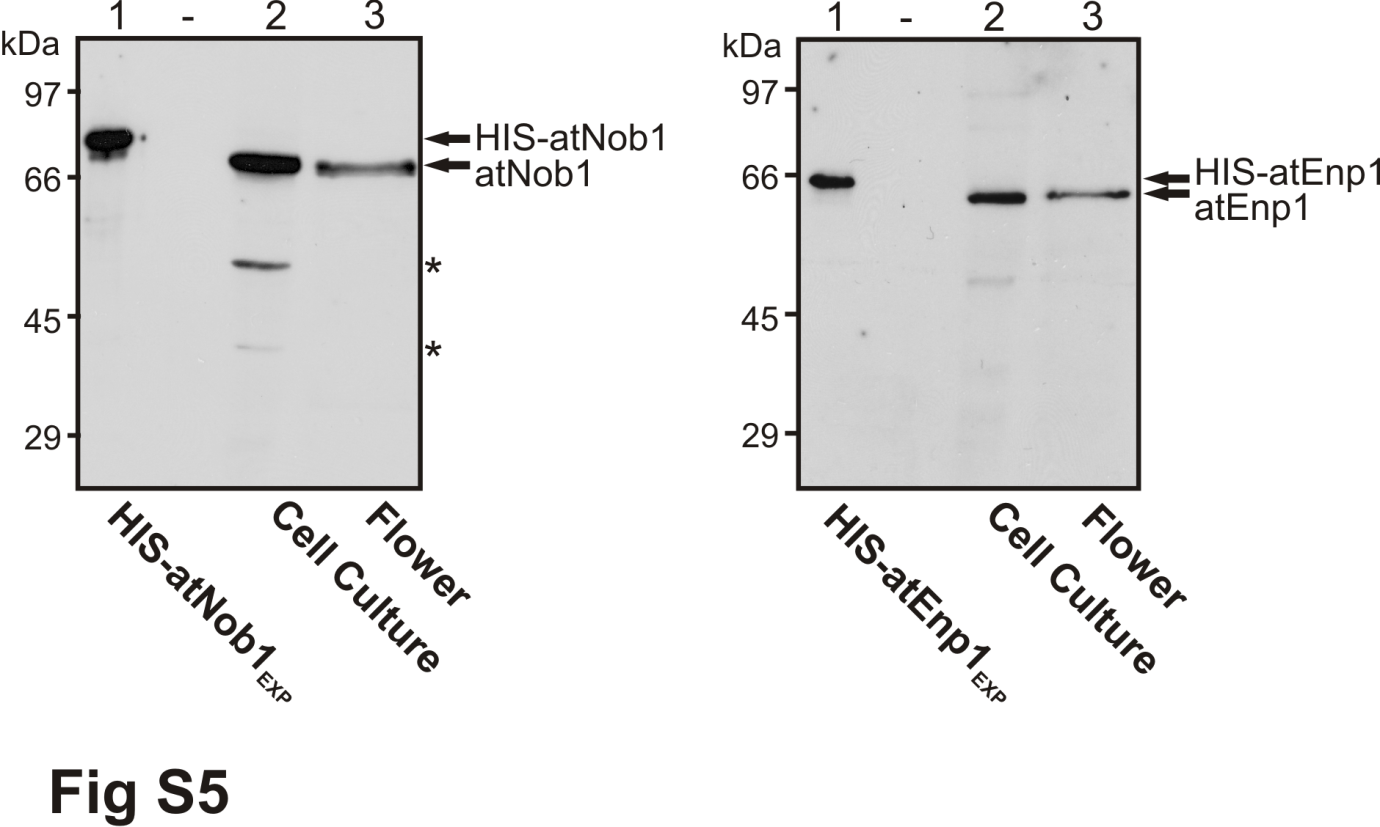

Supplement: Figure S5 — Confirmation of the specificity of the antibodies against atEnp1 and atNob1. (DOCX) [file pone.0054084.s005.docx]
