# Supplementary material for: 40S Ribosome Biogenesis Co-Factors Are Essential for Gametophyte and Embryo Development
Source: PLoS One. 2013 Jan 30;8(1):e54084. doi: 10.1371/journal.pone.0054084 (PMC3559688; doi:10.1371/journal.pone.0054084)

**Figure S6.** Growth analysis of wild-type and heterozygous lines.

All heterozygous insertion lines were grown on MS plates containing the corresponding antibiotic to distinguish plants carrying the T-DNA from wild-type. After two weeks on plates the plants were transferred to soil and after 5 and 9 weeks of plant growth photos were taken. Of each line one representative plant is shown (bottom right). Squares in the background have a size of 3x3 cm. The scale bar for the images of 5 week old plants indicates 1 cm. Please note that the difference in rosette size and inflorescence height between wild-type and *noc4 +/-* or *enp1 +/-* is most likely due to an altered sensitivity of these lines to the antibiotic.


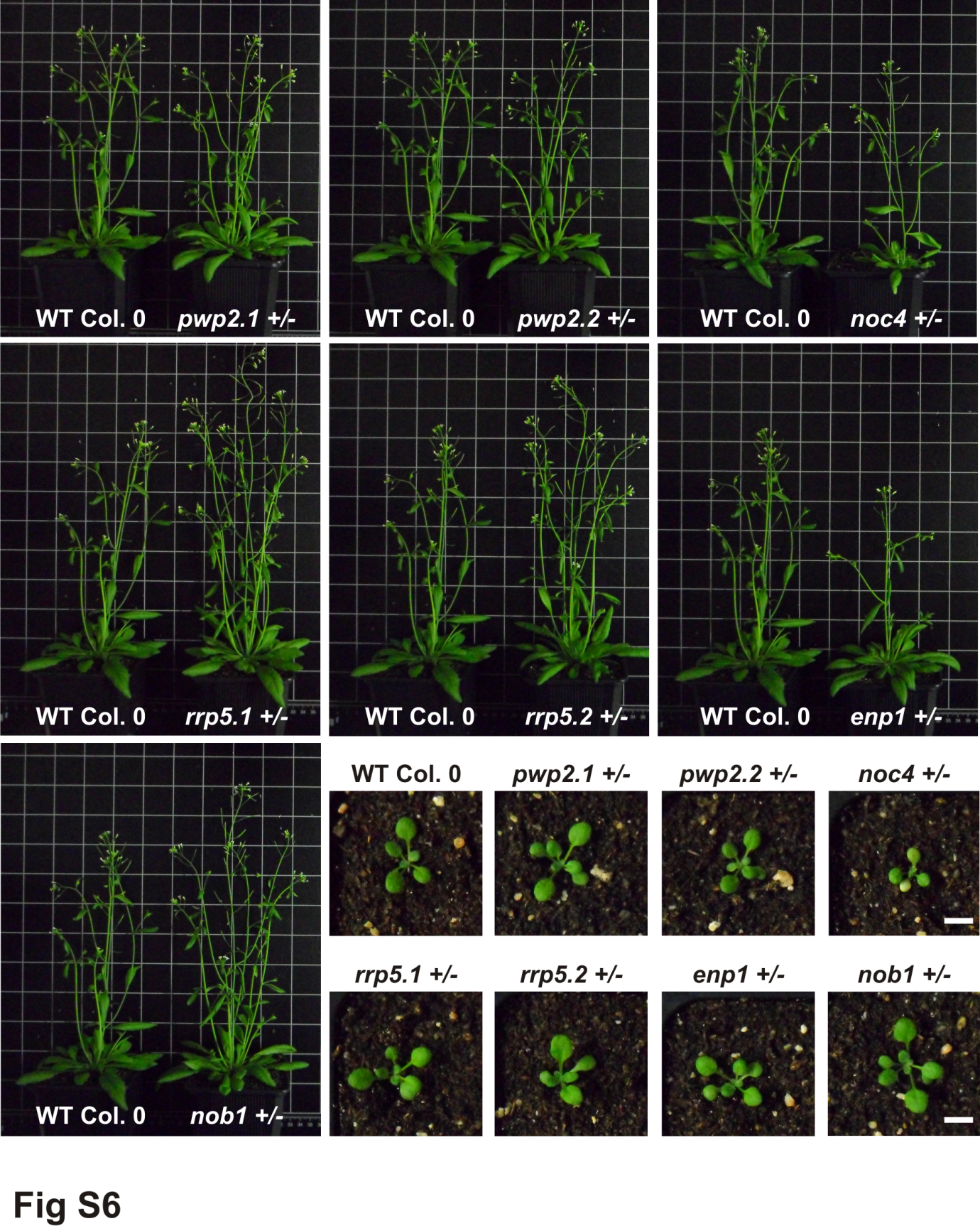

Supplement: Figure S6 — Growth analysis of wild-type and heterozygous lines. (DOCX) [file pone.0054084.s006.docx]
