# Supplementary material for: 40S Ribosome Biogenesis Co-Factors Are Essential for Gametophyte and Embryo Development
Source: PLoS One. 2013 Jan 30;8(1):e54084. doi: 10.1371/journal.pone.0054084 (PMC3559688; doi:10.1371/journal.pone.0054084)

**Figure S7.** Quantification of the P-A3, 20S, 35S and 33S transcript in different genotypes.

The bands of the northern blots shown in Figure 6 were quantified using the phosphor imager. The observed values were background corrected and set into relation to the value observed for 33S or 27S on the same northern blot. Shown are the results for the P-A3 and 20S in relation to 33S and 35S, 33S and 27S in relation to each other. The results for all heterozygous plants are shown.


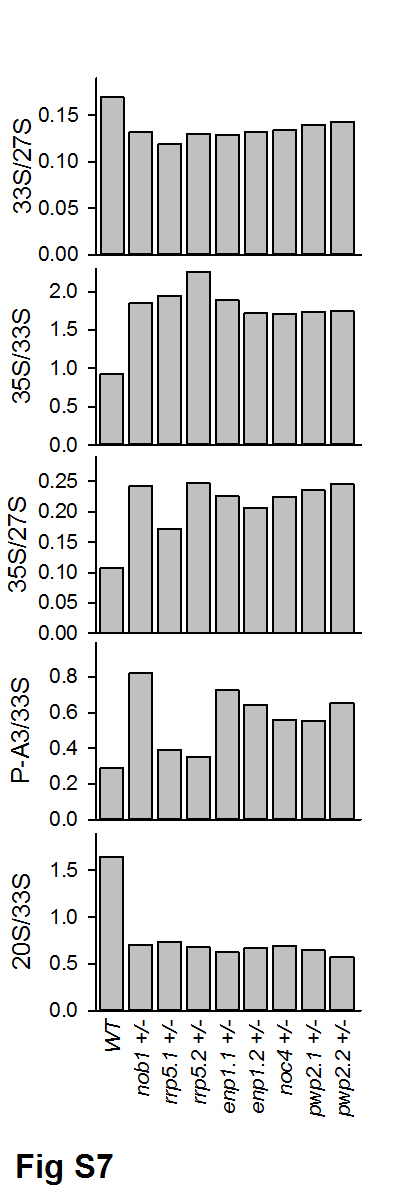

Supplement: Figure S7 — Quantification of the P-A3, P1-A3 and 20S transcript in different genotypes. (DOCX) [file pone.0054084.s007.docx]
