# Supplementary material for: 40S Ribosome Biogenesis Co-Factors Are Essential for Gametophyte and Embryo Development
Source: PLoS One. 2013 Jan 30;8(1):e54084. doi: 10.1371/journal.pone.0054084 (PMC3559688; doi:10.1371/journal.pone.0054084)
Supplement: Figure S8 — Size distribution of siliques from wild-type and heterozygote lines. (DOCX) [file pone.0054084.s008.docx]

**Figure S8.** Size distribution of siliques from wild-type and heterozygote lines.

Siliques from wild-type and heterozygous lines were harvested after 10 weeks and photographed. Siliques of one representative plant are shown. All images are adjusted to the same size and the scale bar shown in the last image indicates 0.5 cm.

**
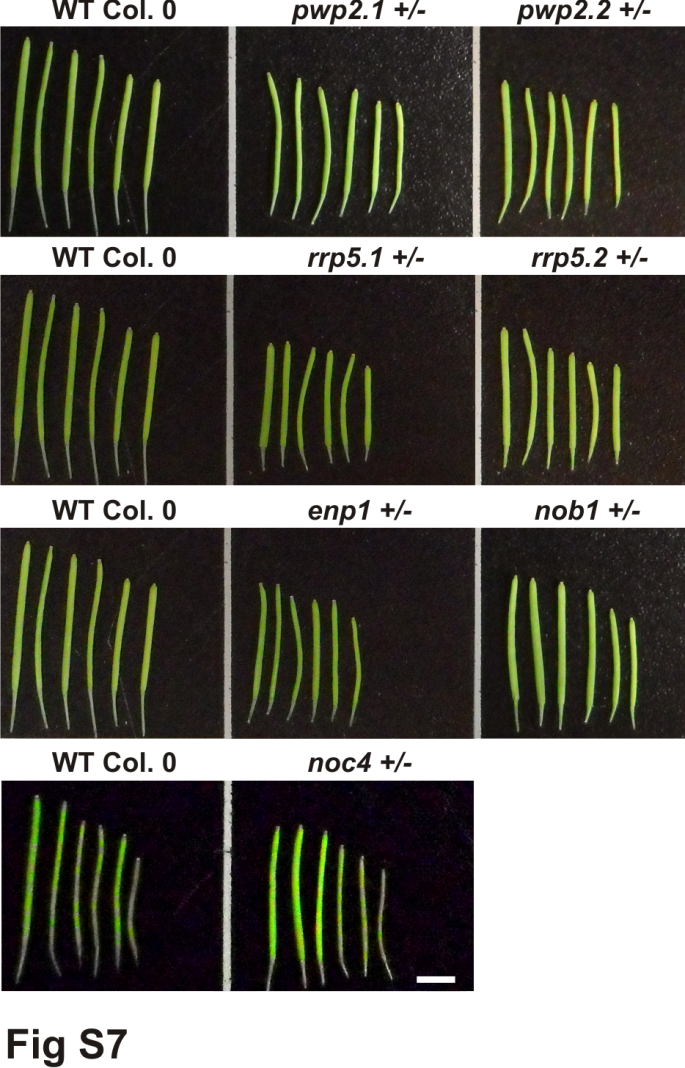
**

**Fig S8**
