# Supplementary material for: 40S Ribosome Biogenesis Co-Factors Are Essential for Gametophyte and Embryo Development
Source: PLoS One. 2013 Jan 30;8(1):e54084. doi: 10.1371/journal.pone.0054084 (PMC3559688; doi:10.1371/journal.pone.0054084)
Supplement: Figure S9 — Properties of the genomic DNA. (DOCX) [file pone.0054084.s009.docx]

**Figure S9.** Properties of the genomic DNA

A, Shown are the alternative gene models (bars) indicating non-coding (white) and coding regions (green). Polymorphisms by substitutions (yellow diamonds), insertion (red diamond) or deletion (blue diamond) are indicated. Position of T-DNA insertions are given by arrowheads. DNA replication origins are shown by blue bars. Y-patch (green half circle) or TSS promoter elements (blue half circle; Yamamoto and Obokata, 2008) and polyadenylation sites (black diamond) are indicated. Regions of DNA methylation (Zhang et al. 2006) are indicated by yellow bars under the gene model. All information’s were extracted from http://gbrowse.arabidopsis.org/cgi-bin/gbrowse/arabidopsis. B, The organization of the DNA (20 kB) surrounding the gene of interest is shown. Natural transposons are marked in orange, DNA replication origins are shown by blue bars and non coding RNAs in violet. Regions of methylation are indicated on top. All information was extracted from http://gbrowse.arabidopsis.org/cgi-bin/gbrowse/arabidopsis.

**
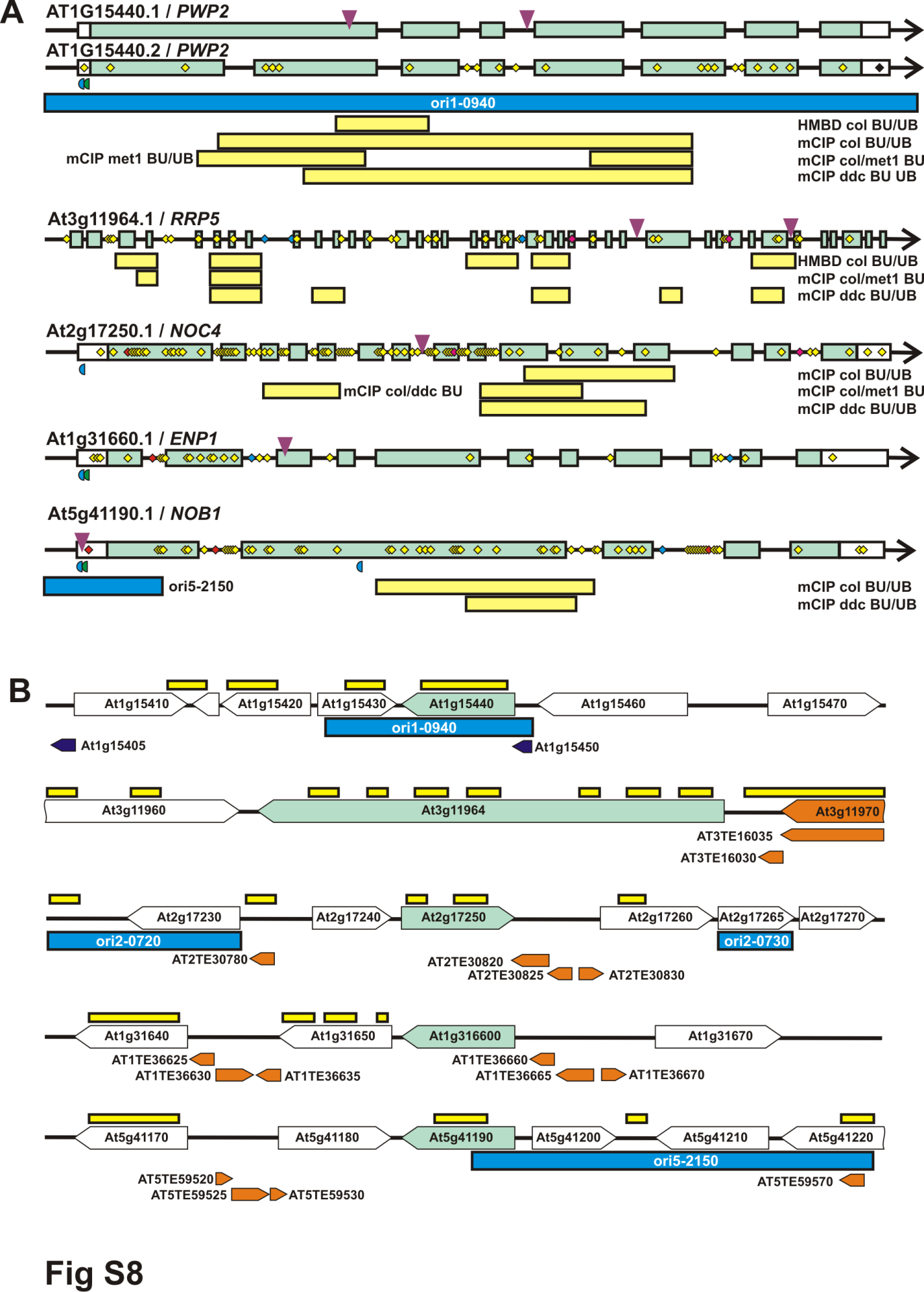
**

**Fig S9**
