# Supplementary material for: 40S Ribosome Biogenesis Co-Factors Are Essential for Gametophyte and Embryo Development
Source: PLoS One. 2013 Jan 30;8(1):e54084. doi: 10.1371/journal.pone.0054084 (PMC3559688; doi:10.1371/journal.pone.0054084)
Supplement: Table S1 — Border sequences of T-DNA insertions. (DOCX) [file pone.0054084.s010.docx]

**Supporting Table S1:** Border sequences of T-DNA insertions

| **Supporting Table S1:** Border sequences of T-DNA insertions | | |
| --- | --- | --- |
| **plant line** | **T-DNA position** | **Sequence** |
| ***pwp2.1*** | 1142 | NNNNNNNNNNGGNNTGANCCAAAGGTCAGCCAATTACCACGCTCATTGAAGACAGCTGTTGTTAGCTTCTGCCTAGAAATAGAGAGCAGATGGATACAGATGAAATCTGGCATCTGATAAAGCCCAAAAACACCATTTGAGAACCCCACCACAACCATGTCAAGTCCCTGATGATAGTCGCAAG |
|  | 1159 | GGTACGTAAATTCCGGACATGAAGCCATTTACAATTGAATATATCCTGGGGCAACTGTTAGTCTGGGATTGGCGAACCTAAACCTATATACTGAAGCAACAAGGACACTATTTTGACGTCAACTGTGTCACTTACTCACCGGATTCGCAGCTCTTGGCAACTGGTGCTG |
|  |  |  |
| ***pwp2.2*** | 1850 | NNNNNNNNNNCGNNNNNANCCNTTTACAATTGAATATACCAATGTATTACACCAATGAATTATTCAGGATATATATAGACTATGACAGTTTCTTTACCGTTAAGGGAGAAAACATCAACCCATGAACAGGAGCCTCATGCCCACTCAGAATATCCTTTATTTGCCCAGTCTTCTTGGACCATACAAAAATCTGCACATAGGCAAGCA |
|  | 1863 | NNNNNNNNTNNNNGNNTGANCCATTTACAATTGAAAGAAGTCAAAATACAATTTGCTTCGTGCCTCTGAACAATTATGTGAACTTCTGCCTTATGGTGTTATAACTTCCATGCCTTATCTAGTTCATATATTTCTTTCTCTGCAGCAACTTTT |
|  |  |  |
| ***rrp5.1*** | 8761 | NNNNNNNNNGNNANNNNNNCACCCCAGTACATTAAAAACGTCCGCAATGTGTTATTAAGTTGTCAATAAGCGTCAATTTGTTCATATATCACCATCCCACAAGTTGCTAGAATAGACTGACTAAAACCGTTTTAAACCCTAAGGGTATGTGACTGTA |
|  | 8762 | NNNNNNNNNNNGNNAAGAAACCACCCCAGTACATTAAAAACGTCCGCAATGTGTTATTAAGTTGTCTAAGCGTCAATTTGTCCTGGTGTAATAATTTTATTCTACGTGAATTATCTTATTTAGGATTTCCTCTAGCTATTATGTTTTTCAAAA |
|  |  |  |
| ***rrp5.2*** | 11209 | NNNNNNTNNCNNNNTGANCCNTTTACAATTGTGAAGCCAAACTTTGTTTCTAAAGAAATAACCAACCTTTCCTCTTTGTCTTTTTGCTTTTCCCTTCTCTTGCTCTTTTCATCTTTATTGGCTCCCAGTAGTTTCTCCTGATTCTGGCTGCTGTCAAAGTCCGTCTCCTCAATGTCATCAAGGTCAACCTCAAGCGGAGGGATAGAAGCCCTTGATTCTACTTGGGCAAGAA |
|  |  |  |
| ***noc4*** | 1511 | NNNNNNNNNNNNNNNNNNAAACCACCCCAGTACATTAAAAACGTCCGCAATGTGTTATTAAGTTGTCTAAGCGTCAATTTGTTTACACCACAATGTCTAAACCTATTCACAGATTACGGATTCAGTATATTCAGCAAACTTGCAACTGGAAACTTACAGTGCTGTCTCCTTTCTCAGTTTTCTTCTTTTTATCTGTCGGCTTCTCACTAATACTTTCATCTGCACAATAACTCTTATTAGCAAATCTAAACACATAGG |
|  | 1512 | NNNNNNNNNGNNAAGNNANNACCCCAGTACATTAAAAACGTCCGCAATGTGTTATTAAGTTGTCTAAGCGTCAATTTGTTTACACCGTCAATGTGAATAGGTTTTCTTCCTGGCATGGAATTCCTTACATTTGCCTCTTTATTTATTCTGTTAAAACTCCAGCTCCTTTCTCCGA |
|  |  |  |
| ***enp1*** | 653 | NNNNNNNNNNNNNGNACTTNCTTTTATAATAACGCTGCGGACATCTACATTTTTGAATTGAAAAAAAATTGGTAATTACTCTTTCTTTTTCTCCATATTGACCATCATACTCATTGCTGATCCATGTAGATTTCCCGGACATGAAGCCATTTACAATTGAATATCCTCATTAATCTCCTCCTAATATAAGTTACATAACAATATCAAAACATAAACATGAAANACTCAATTTTGGCATCGTTCTAAGCAATTCAATCTAGTGGATCCCCCGGGCTGCANGANTTCAAGATCTGGCTTATCTATTCC |
|  |  |  |
| ***nob1*** | 2 | NNNNNNNNNNNNNNGNNNNNNNNNCACCCCAGTACATTAAAAACGTCCGCAATGTGTTATTAAGTTGTCTAAGCGTCAATGGGTTTTTGTTTACACTCTTCTTCTTCTCTCCATCTCTCAGTCTCTTCGTTAAACCCACCCACATTTGAAGCGGCAAATAACGAAAACCATGGATCCGAAACCAACCTCGATGTGGAGCTCAATTGTGAAGAAAGATCCACCTTCAA |
| Given is the gene name, the positioning of the T-DNA boarder within the gene and the sequence obtained by sequencing. Underlined sequence refers to the T_DNA specific sequence. | | |
