# Supplementary material for: 40S Ribosome Biogenesis Co-Factors Are Essential for Gametophyte and Embryo Development
Source: PLoS One. 2013 Jan 30;8(1):e54084. doi: 10.1371/journal.pone.0054084 (PMC3559688; doi:10.1371/journal.pone.0054084)
Supplement: Table S2 — Oligonucleotides used in this study. (DOCX) [file pone.0054084.s011.docx]

**Supporting Table S2:** Oligonucleotides used in this study.

| **Supporting Table S2:** Oligonucleotides used in this study. | | | | |
| --- | --- | --- | --- | --- |
| **gene name** | **oligonucleotide** | | **application** | **sequence** |
| **atPWP2** | 1 | *pwp2.1* FP | PCR | GTTGGTACTGAGCTTAAGAAA |
| (At1g15440) | 2 | *pwp2.1* RP | PCR | TCTTCTTGGACCATACAAAAA |
|  | 3 | *pwp2.2* FP | PCR | gcactgttcgtgcttgggat |
|  | 4 | *pwp2.2* RP | PCR | aaaccggcgcagaagaacct |
|  | 5 | PWP2 FP | qRT-PCR | TTGACGATACCTTCATCTTTGATCC |
|  | 6 | PWP2 RP | qRT-PCR | TTGACGATACCTTCATCTTTGATCC |
| **atRRP5** | 1 | *rrp5.1* FP | PCR | acagtcacatacccttaggg |
| (At3g11964) | 2 | *rrp5.1* RP | PCR | atctaatggatcagggaccc |
|  | 3 | *rrp5.2* FP | PCR | cggaatgaagagttcctatc |
|  | 4 | *rrp5.2* RP | PCR | atttatagtcctcaaggccc |
|  | 5 | RRP5 FP | qRT-PCR | ATATTGAGAAAGCCAGGTCTATTGC |
|  | 6 | RRP5 RP | qRT-PCR | TCCATGTTCATTTTCCAAATTGAAG |
| **atNOC4** | 1 | *noc4* FP | PCR | gattcactctgaggttgata |
| (At2g17250) | 2 | *noc4* RP | PCR | cggagaaaggagctggagtt |
|  | 5 | NOC4 FP | qRT-PCR | GATGTGTACAAGGAGGTTCTTGCCA |
|  | 6 | NOC4 RP | qRT-PCR | ACACCCCCAATGTCGTACGATTTTG |
| **atENP1** | 1 | *enp1* FP | PCR | gcagttgattgaagctggta |
| (At1g31660) | 2 | *enp1* RP | PCR | atatcctctctcactcgcgg |
|  | 5 | ENP1 FP | qRT-PCR | TGATTTGGCACCAGTCTCTTCTTAC |
|  | 6 | ENP1 RP | qRT-PCR | AACAAGATGGTGTTTTTGCCTCTGG |
| **atNOB1** | 1 | *nob1* FP | PCR | gtctcttcgttaaacccacc |
| (At5g41190) | 2 | *nob1* RP | PCR | ggtggaggaacatctctaag |
|  | 5 | NOB1 FP | qRT-PCR | GCATGTAAACCACGAATTACACTTC |
|  | 6 | NOB1 RP | qRT-PCR | GGAGTTGATCTTCCCGTAGAATCAG |
| **atUBI3** | 1 | UBI3 FP | qRT-PCR | GTAAGCAGCTCGAAGATGGACGCAC |
| (At5g03240) | 2 | UBI3 RP | qRT-PCR | AGGGTCTTCACAAAGATCTGCATAC |
| **atACT2** | 1 | ACT2 FP | qRT-PCR | ATGTCGCCATCCAAGCTGTTCTCTC |
| (At3g18780) | 2 | ACT2 RP | qRT-PCR | GAGAAACCCTCGTAGATTGGCACAG |
|  |  | RT T24VN | RT | TTTTTTTTTTTTTTTTTTTTTTTTVN |
| Given is the gene name and corresponding identifier in Arabidopsis, the oligonucletide by number and name, the application (qRT-PCR, PCR, RT) and the sequence. | | | | |
