# Supplementary material for: 40S Ribosome Biogenesis Co-Factors Are Essential for Gametophyte and Embryo Development
Source: PLoS One. 2013 Jan 30;8(1):e54084. doi: 10.1371/journal.pone.0054084 (PMC3559688; doi:10.1371/journal.pone.0054084)
Supplement: Table S3 — Probes for northern blot analysis. (DOCX) [file pone.0054084.s012.docx]

**Supporting Table S3:** Probes for northern blot analysis

| **Supporting Table S3:** Probes for northern blot analysis | |
| --- | --- |
| **probe no.** | **sequence** |
| **p1** | CCTAGGCGGATCCATGCTTTCCAAC |
| **p2** | ACGGCAATTCCCCGCCACATCC |
| **p3** | GGTCGTTCTGTTTTGGACAGGTATC |
| **p4** | CGTTTTAGACTTCAGTTCGCAG |
| **p5** | GGATGGTGAGGGACGACGATTTGTG |
| **p6** | CGTTAAGGAGCTGTTGCTTTGTTAGTGTAG |
| **p23** | GTTCCAACTACTCTACCGAAGTAC |
| **7SL** | ACTGGGCAGCCCAGAAACATGC |
| **eEF1**α | AGACACCTCCTTGATGATTTCATCG |
| Given is the probe number for northern blot analysis and the sequence. | |
