# Supplementary material for: 40S Ribosome Biogenesis Co-Factors Are Essential for Gametophyte and Embryo Development
Source: PLoS One. 2013 Jan 30;8(1):e54084. doi: 10.1371/journal.pone.0054084 (PMC3559688; doi:10.1371/journal.pone.0054084)
Supplement: Table S4 — Statistical evaluation of transmission rates. (DOCX) [file pone.0054084.s013.docx]

**Supporting Table S4.** Statistical evaluation of transmission rates.

| **Supporting Table S4:** Statistical evaluation of transmission rates. | | | | | | | | | | | | |
| --- | --- | --- | --- | --- | --- | --- | --- | --- | --- | --- | --- | --- |
| **plant line** | **selfing** | | |  | **paternal backcrossing** | | |  | **maternal backcrossing** | | | **segregation pattern** |
|  | **TR** | **H_0_ = 1; H_0_ = 2** | **MLH*** |  | **TR** | **H_0_ = 0; H_0_ = 1** | **MLH*** |  | **TR** | **H_0_ = 0; H_0_ = 1** | **MLH*** |  |
|  | [AB_r_/AB_s_] | p(H_0_=1)/p(H_0_=2) | H_0_ = |  | [AB_r_/AB_s_] | p(H_0_=0)/p(H_0_=1) | H_0_ = |  | [AB_r_/AB_s_] | p(H_0_=0)/p(H_0_=1) | H_0_ = |  |
| ***pwp2.1 +/-*** | 0.63 ± 0.15 | 2.85e+5 | 1 |  | 0.66 ± 0.19 | 1.02e-2 | 1 |  | 0.00 ± 0.00 | + ∞ | 0 | female gametophyte mutation |
| ***pwp2.2 +/-*** | 0.56 ± 0.23 | 3.86e+7 | 1 |  | 0.88 ± 0.23 | 7.28e-5 | 1 |  | 0.01 ± 0.02 | 4.44e+22 | 0 | female gametophyte mutation |
| ***noc4 +/-*** | 1.95 ± 0.32 | 3.02e-7 | 2 |  | 1.01 ± 0.16 | 1.33e-13 | 1 |  | 1.03 ± 0.20 | 1.83e-14 | 1 | Mendelian segregation |
| ***rrp5.1 +/-*** | 0.80 ± 0.24 | 2.56e+7 | 1 |  | 0.82 ± 0.22 | 1.54e-8 | 1 |  | 0.02 ± 0.03 | 1.04e+6 | 0 | female gametophyte mutation |
| ***rrp5.2 +/-*** | 0.40 ± 0.17 | 4.02e+5 | 1 |  | 0.72 ± 0.25 | 5.35e-8 | 1 |  | 0.00 ± 0.00 | + ∞ | 0 | female gametophyte mutation |
| ***enp1 +/-*** | 0.03 ± 0.02 | 7.42e+1 | 1 |  | 0.01 ± 0.03 | 7.99e+7 | 0 |  | 0.00 ± 0.00 | + ∞ | 0 | male and female gametophyte mutation |
| ***nob1 +/-*** | 1.00 ± 0.34 | 1.30e+4 | 1 |  | 0.35 ± 0.16 | 1.41e+2 | 0 |  | 0.07 ± 0.10 | 6.32e+10 | 0 | male and female gametophyte mutation |

For the transmission rates of selfing, paternal and maternal backcrossing the corresponding p-values were calculated. For selfing the null hypothesis was either H_0_ = 2 or H_0_ = 1, for paternal and maternal backcrossing the null hypothesis was either H_0_ = 1 or H_0_ = 0. The alternative hypothesis (H_1_) was assumed as H_1_ ≠ H_0_. Because the determined transmission rates were not ideal the ratio between the two p-values (H_0_=1 or H_0_=2; H_0_=0 or H_0_=1 for selfing or maternal/paternal backcrossing, respectively) is presented to judge which transmission is more likely. *MLH…most likely hypothesis
